# Multiscale Organization of Neural Networks in a 3D Bioprinted Matrix

**DOI:** 10.1002/advs.202504455

**Published:** 2025-05-28

**Authors:** Huiyu Yang, Jiangang Zhang, Yiran Li, Zihan Zhong, Wenhua Li, Haojun Luo, Yanyong Liu, Liujian Ouyang, Zhuoran Jiang, Yuning Sun, Hang Sun, Lulu Liu, Huayu Yang, Yu Wang, Nan Yang, Wenbin Ma, Yilei Mao

**Affiliations:** ^1^ Department of Neurosurgery PUMCH PUMC & CAMS Beijing 100730 China; ^2^ Department of Liver Surgery PUMCH PUMC & CAMS Beijing 100730 China; ^3^ Institute of Clinical Medicine Translational Medicine Center PUMCH PUMC & CAMS Beijing 100730 China; ^4^ Eight‐Year Medical Doctor Program CAMS & PUMC Beijing 100730 China; ^5^ Department of Pharmacology Institute of Basic Medical Sciences CAMS & PUMC Beijing 100005 China; ^6^ Department of Endocrinology Children's Hospital of Zhejiang University School of Medicine National Clinical Research Center for Child Health Hangzhou Zhejiang 310003 China; ^7^ Center for Biomedical Technology of National Infrastructures for Translational Medicine State Key Laboratory of Complex Severe and Rare Diseases in Peking Union Medical College Hospital Beijing 100730 China

**Keywords:** 3D bioprinting, CNS disease, neural network, primary neuron model

## Abstract

The efficient establishment of in vitro neural models that accurately mimic the structural and functional connectivity of neural networks is critical in neuroscience research. 3D bioprinting shows great potential for constructing sophisticated in vitro models with high freedom of design. However, mature neurons are delicate and susceptible to manipulation. Here, extrusion‐based 3D bioprinting is employed to fabricate gelatin methacryloyl (GelMA)‐based constructs containing embryonic day 18 (E18) rat cortical neurons, referred to as 3D neuMatrix. 3D neuMatrix displays favorable neuronal viability, with the progressive formation of a 3D brain‐like neural network with local and long‐range functional axon connections. Compared with 2D cultured neurons, 3D neuMatrix is more similar to the E18 cortex according to the bulk transcriptomic profile, with a recreation of cellular components in the cerebral cortex. The 3D neuMatrix is employed to establish a disease model of ischemic stroke, with a faithful recapitulation of the viability, function, and transcriptomic features of rats with middle cerebral artery occlusion/reperfusion (MCAO/R). These findings demonstrate the formation of multiscale neural circuits within 3D neuMatrix and its valuable potential in the study of neurodevelopment, disease modeling with drug screening, and in vitro intelligence.

## Graphical abstract

A 3D bioprint model of primary neurons has been engineered with a millimeter‐scale functional neural network, and it recapitulates in vivo transcriptomic features under both normal and disease conditions to the greatest extent. The successful integration of mature neurons and 3D bioprinting signifies a major advance in neuroscience modeling, facilitating mechanistic research and therapeutic development for CNS disorders.

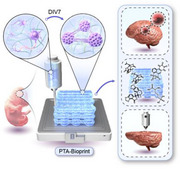

## 1 Introduction

The brain is a structurally complex organ composed of various highly specialized cells that are interconnected to exercise sophisticated neural functions.^[^ ^1^ ^]^ Primary neural cells are essential models for investigating the structural and functional basis of neural circuits and can help elucidate the fundamental principles of neurobiology and the pathogenesis of central nervous system (CNS) disorders, such as Alzheimer's disease.^[^ ^2^, ^3^ ^]^ Compared to animal models or pluripotent stem cell‐derived models, primary neural cells allow more precise control of experimental conditions and retain the biological characteristics of the parent tissue.^[^ ^4^ ^]^ Additionally, these models are extensively used to assess the pharmaceutical effects on neural activity, providing crucial support for drug development.^[^ ^5^ ^]^ However, traditional primary neural cells are cultured in 2D polystyrene dishes, lacking the complexity of neural networkstructures brought by the third dimension and proper mechanical stimulation from the extracellular matrix (ECM), which influences circuit formation and neural function.^[^ ^6^ ^]^ Fabrication of 3D artificial neural tissue by employing advanced bioengineering technologies such as scaffold‐based cultures,^[^ ^7^ ^]^ neural spheroids/organoids,^[^ ^8^ ^]^ and brain‐on‐a‐chip^[^ ^9^ ^]^ models shed light on these limitations.

3D bioprinting is an emerging biofabrication technology for constructing functional tissues with predesigned cellular components and biophysical properties. Among different 3D bioprinting methodologies, extrusion‐based 3D bioprinting is a high‐throughput and convenient method for producing 3D tissue with prominent freedom of design and has been widely used in cancer biology and regenerative medicine.^[^ ^9^, ^10^, ^11^, ^12^ ^]^ Compared to other models, 3D bioprinted neural models offer various advantages, including precise cellular placement and structural fidelity, such as the layered arrangement of neurons within the cerebral cortex and the directionality of neuronal axons and dendrites,^[^ ^13^, ^14^ ^]^ as well as reproducibility and cost‐effectiveness for accurate disease modeling, standardization, and analysis. However, due to the delicacy of primary neural cells, appropriate rheological properties of biomaterials and optimal printing parameters are essential for producing highly functional neural networks. Previous attempts at 3D bioprinting of primary neural cells received low cell viability and noncomprehensive analyses.^[^ ^15^, ^16^, ^17^ ^]^ Despite years of advancement in bioprinting technology, the construction of functional primary neural tissues with multiscale organization of neural networks remains a major obstacle. We hereby optimized the printing parameters and model design based on previous experience in bioprinting functional liver tissue and primary tumor‐derived tissue to reduce shear force damage to cells during printing.^[^ ^10^, ^11^, ^12^ ^]^ We explored the optimal bioink composition containing gelatin methacryloyl (GelMA), which is a gelatin‐based biomaterial with good biocompatibility, excellent stability, and adjustable biomechanical properties,^[^ ^18^ ^]^to facilitate the survival, maturation, and formation of synaptic connections of primary neural cells in vitro.

**Figure 1:**
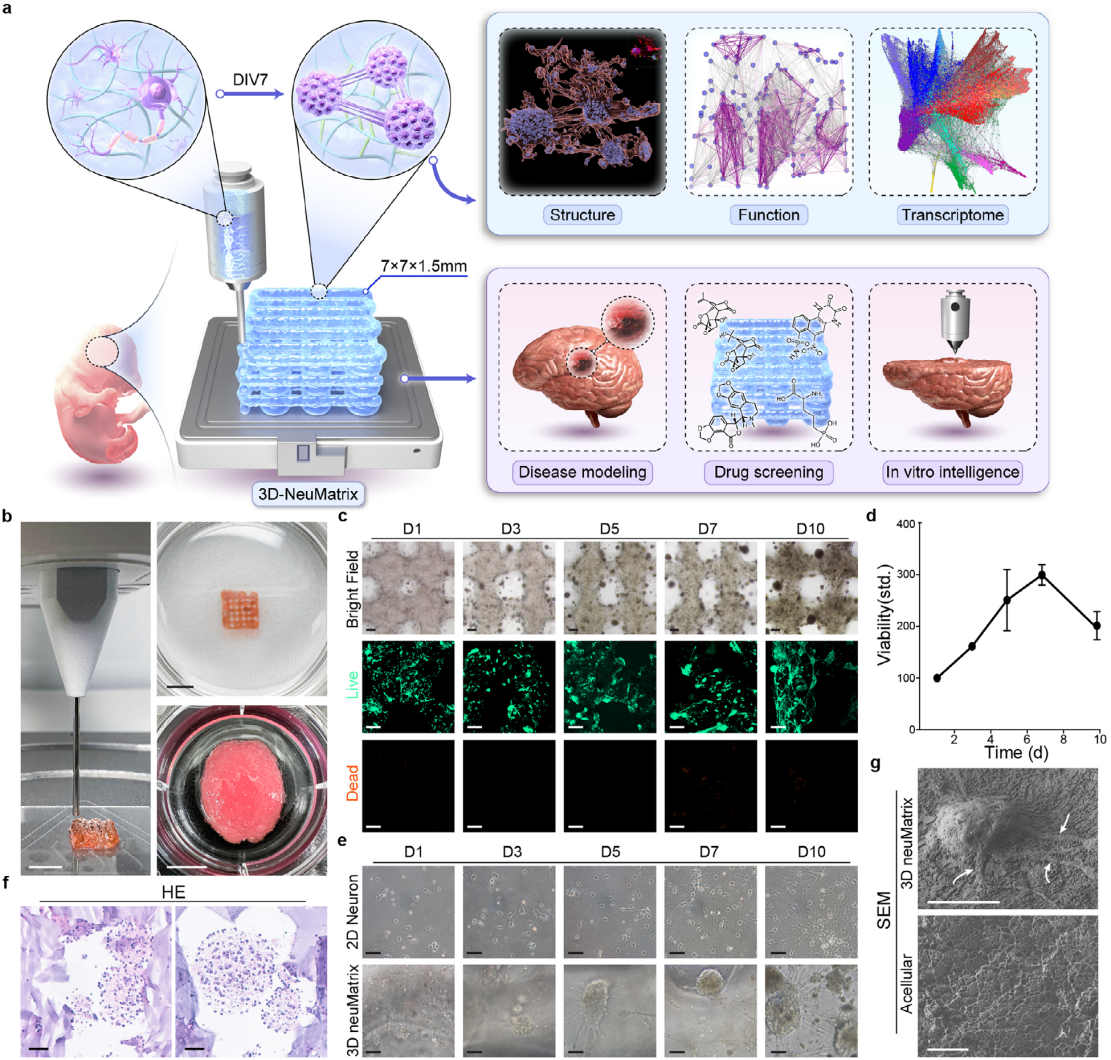
Fabrication of the 3D neuMatrix. a) Schematic illustration of 3D neuMatrix construction with high structural, functional, and transcriptomic fidelity and potential employment in disease modeling, drug screening, and in vitro intelligence. b) Photographs of the bioprinting process (left), 3D neuMatrix (upper right), and mini‐brain constructed by floating bioprinting (lower right). c) Bright field and Live/Dead staining image sequences at DIV 1, 3, 5, 7, and 10. d) Viability of the 3D neuMatrix as determined by ATP quantification during in vitro culture. e) Comparison of 2D neuron and 3D neuMatrix structural transformation during in vitro culture. f) HE staining of assembled cell clusters with various diameters within the 3D neuMatrix. g) SEM images of cell clusters within a 3D neuMatrix (upper) marked with protrusions (arrow) and acellular GelMA ECM (lower). Scale bars, 5 mm (b), 200 µm (c), and 50 µm (e–g).

In this study, we described 3D neuMatrix, a novel 3D bioprinted primary neural tissue that features complex local and large‐scale functional neural network formation. We validated the biological fidelity of 3D neuMatrix in both physiological and pathological states through next‐generation sequencing, with single‐cell analysis demonstrating that 3D neuMatrix can accurately recapitulate various neuron subtypes and gliogenesis in the cerebral cortex. The 3D neuMatrix provides a novel platform for understanding neural circuits, disease modeling, drug screening, and the evolution of in vitro intelligence^[^ ^19^, ^20^ ^]^ (**Figure** **1**a).

## 2 Results

### 2.1 Key Biological Properties of the 3D neuMatrix

Primary cortical cells from E18 rat embryos were isolated and mixed with GelMA and lithium phenyl‐2,4,6‐trimethylbenzoylphosphinate to the final concentration of 15 million cells per milliliter (1.5 × 10^7^ cells mL^−1^), simulating the cellular density within cortical tissue. The hydrogel containing primary cortical cells was loaded onto an extrusion‐based 3D bioprinter and fabricated in a layer‐by‐layer manner (Figure 1b). For 3D neuMatrix used in the subsequent in vitro experiments, a 7×7×1.5 mm 6‐layered rigid cuboid structure was bioprinted (Figure 1b). The degree of substitute and concentration of GelMA were optimized according to the viability of primary neural cells during culture and rheological properties of the bioink (Figures S1–S3, Supporting Information). 3D neuMatrix contains interconnected channels with a diameter of ≈500 µm to facilitate the exchange of oxygen and nutrients. After a series of experiments to optimize the bioprinting conditions for 3D neuMatrix (Figure S2, Supporting Information), we have chosen a 21G nozzle with an inner diameter of 510 µm to reduce the shear force during the manufacturing process while ensuring bioprinting resolution. The bioprinted constructs were crosslinked under 405 nm light of 7.5 mW cm^−2^ for 10 s to form a 3D neuMatrix with a stable structure throughout long‐term in vitro culture without damaging cortical cells. Moreover, it is possible to bioprint any intricate structure with high spatial resolution under the above printing conditions. For example, by adopting a self‐healing hydrogel bath as a printing chamber, an in vitro mini‐brain was fabricated by floating bioprinting and washout (Figure 1b), laying the groundwork for future brain tissue engineering.

The primary neural cells in the 3D neuMatrix were highly viable after bioprinting and during in vitro culture, with only a small amount of apoptosis detected at 7 days in vitro (DIV) by propidium iodide staining (Figure 1c). ATP quantification confirmed that the cell viability gradually increased during the first week, reaching a maximum value of 299 ± 20% at DIV 7, and slightly decreased in the subsequent culture but remained at a relatively high level (201 ± 27% at DIV 10) (Figure 1d). 3D neuMatrix even maintained viability during prolonged culture for up to 28 days (Figure S1a,b, Supporting Information). The microscopic image sequence demonstrated self‐aggregation of cell clusters with interconnected structures in the 3D neuMatrix (Figure 1c,e), with a tendency toward the surface of 3D construct, probably due to the accessibility of nutrients, but there was still abundant cell networks observed inside the hydrogel, emphasizing its biocompatibility. Compared with 3D neuMatrix, 2D cultured neurons exhibited a random distribution of cells and unorganized neural connections (Figure 1e). Overall, primary neural cells can tolerate the bioprinting process and long‐term in vitro culture, with only a small amount of apoptosis in the centers of cell clusters at the later stages of culture, presumably due to the increased diameter of cell clusters and low oxygen infiltration.^[^ ^21^ ^]^ To investigate the microarchitecture of 3D neuMatrix, hematoxylin‐eosin (HE) staining and scanning electron microscopy (SEM) were employed to visualize cell clusters and the internal structure of the hydrogel. The hydrogel in 3D neuMatrix exhibited a loose and porous framework in which primary neural cells assembled into clusters of diverse sizes in Figure 1f, the largest of which exceeded 200 µm in diameter, with extending neurites formed connections with other cell clusters within the hydrogel (Figure 1g).

### 2.2 Multiscale Neural Networks Within the 3D neuMatrix

**Figure 2:**
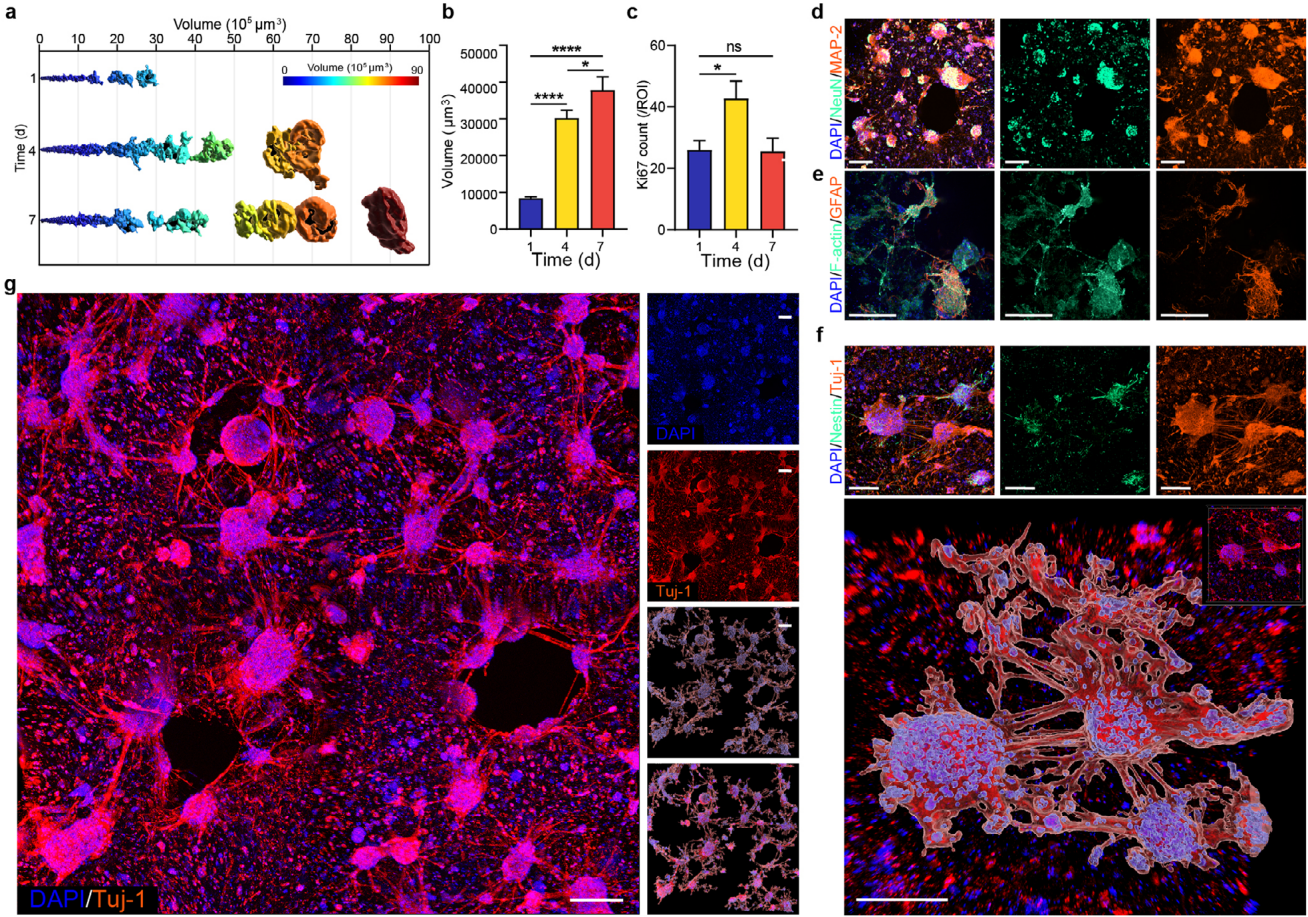
Distinct neural network structure within the 3D neuMatrix. a–c) Representative distribution of neural cluster volume measured by the surface tool of Imaris (a) and the average volume of neural clusters (b) at DIV 1, 4, 7 (n = 4 for each time point). c) Count of Ki67‐positive cells per image taken at 10x magnification (n = 4 for each timepoint). d, e) IF images of DAPI (blue, in all images) for cell nuclei, NeuN (green) and MAP2 (red) for neurons (d), and phalloidin (green) for actin and GFAP (red) for astrocytes (e). f) IF images of Nestin (green) for stemness and Tuj1 (red) for neuron projections (upper), with 3D surface reconstruction of neural clusters and interconnection by Imaris. g) Large‐scale IF image of DAPI (blue) and Tuj1 (red) with neural network reconstruction by Imaris (lower right). Scale bars, 200 µm. The error bars represent the s.e.m., and the P value was calculated by Welch's *t*‐test. ns not significant, ^*^ *P* < 0.05, ^****^ *P* < 0.0001.

To further inquire about the proliferative capacity of 3D neuMatrix, we measured the growth of cell clusters and counted the number of Ki67+ cells during in vitro culture (**Figure** **2**a–c; Figure S4a, Supporting Information). The shape and volume of the cell clusters were calculated by surface analysis of Imaris software. The distribution of cell clusters by volume during in vitro culture is illustrated in Figure 2a. The average volume of cell clusters increased with time, with the most significant increase occurring during the first 3 days as the volume tripled from 8408 to 30215 µm^3^ (Figure 2b). The count of Ki67+ cells per region of interest (ROI) also peaked at DIV 4 (42.8 ± 5.6/ROI) and decreased at DIV 7 to 25.5 ± 4.3/ROI (Figure 2c; Figure S4a, Supporting Information), which correlated with the growth curve of cluster volume and overall viability of 3D neuMatrix (Figure 1c,d).

To investigate the cellular components of 3D neuMatrix at DIV 7, we stained the 3D neuMatrix with specific markers of neurons and astrocytes. Most of the cells inside these cell clusters of various sizes were NeuN+ neurons, with numerous MAP2+ projections within and between cell clusters in all directions (Figure 2d,e; Figure S4b, Supporting Information). Deep staining of SYN indicated the formation of abundant synaptic complexes within neural clusters (Figure S4b, Supporting Information). Nestin+ stem‐like cells were scarcely found within 3D neuMatrix, suggesting that most of the neurons were relatively mature at DIV 7 (Figure 2d; Figure S4b, Supporting Information), consistent with previous findings on the overall growth of 3D neuMatrix. As we had removed most of the white matter during the isolation of primary cells and used serum‐free Neurobasal medium, GFAP+ astrocytes were not in great numbers compared to neurons, even though astrocytes were not screened during the fabrication of 3D neuMatrix due to differences in adherence. Intriguingly, the remaining astrocytes formed a scaffold‐like structure within neural clusters, with long projections along neurites between clusters (Figure 2e; Figure S4b, Supporting Information). This intimacy in structure emphasized the importance of astrocytes in supporting normal neural functions.^[^ ^22^ ^]^ For better visualization of the neural network, we employed Imaris to reconstruct the fiber connections between neural clusters (Figure 2f,g; Video S1, Supporting Information). Locally, the 3D neuMatrix formed a complicated neural network with multiple branching neurites between proximal clusters (Figure 2f). At the millimeter scale of the whole 3D neuMatrix, a 3D neural web was developed with sprouted neural clusters and projections, which highly resembled the hubs and projections of the brain in vivo^[^ ^23^ ^]^ (Figure 2g).

### 2.3 Functional Interconnection and Drug Responsiveness of the 3D neuMatrix

**Figure 3:**
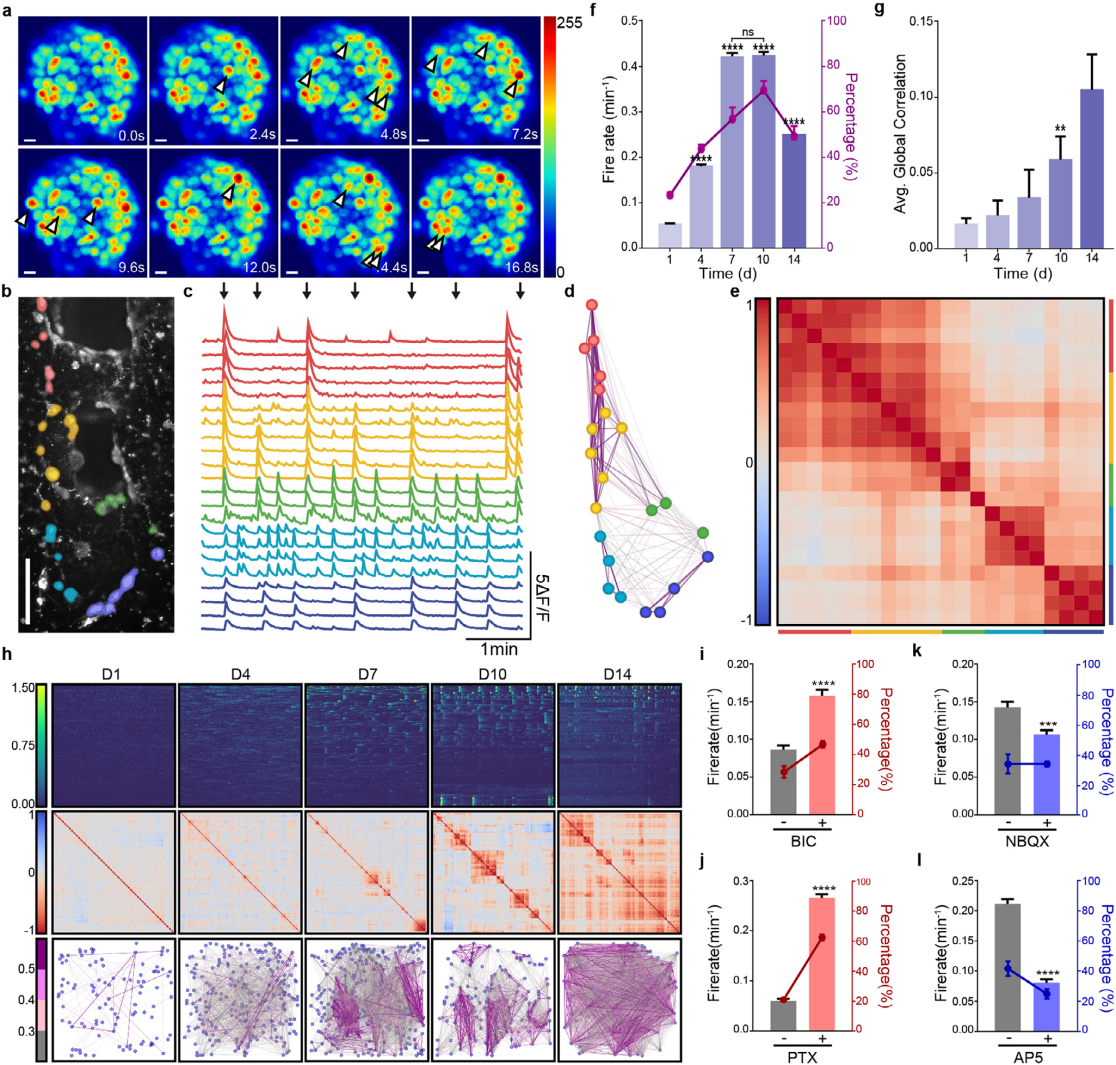
Functional dynamics of the neural network within 3D neuMatrix. a) Image sequence of the calcium signal inside the neural cluster, with a time interval of 2.4 s and white arrows indicating the onset of neuronal firing. b–e) Mesoscopic calcium signal among neural clusters marked in (b), traces of which are illustrated in (c), with black arrows indicating synchronized firing involving ≥3 groups of neural clusters. The correlations of signaling between clusters were portrayed in situ (d) and in matrix (e) in the order of upper to lower position within each group of neural clusters in (b). f) Average firing rate of neural clusters and percentage of firing neural clusters during 5‐min sampling (*n* = 5). g) Average global correlation during in vitro culture (*n* = 5). h) Representative images of the firing matrix (upper), correlation matrix (middle), and correlation diagram (lower) at DIV 1, 4, 7, 10, and 14. i–l) Alteration of the firing rate of neural clusters and percentage of firing neural clusters during 5‐min sampling (n = 3) before and after treatment with 10 µm BIC (i), 50 µm PTX (j), 5 µm NBQX (k), and 50 µm AP5 (l). Scale bar, 10 µm (a), 500 µm (b). The error bars represent the s.e.m., and the P value was calculated by Welch's *t*‐test. ns, not significant; ^**^ *P* < 0.01; ^***^ *P* < 0.001; ^****^ *P* < 0.0001.

After the establishment of large‐scale structural connectivity in the 3D neuMatrix, we assessed the neural function by visualizing calcium oscillations in 3D neuMatrix using Fluo‐4 AM. At the microscopic scale, neurons within the cluster exhibited independent oscillations, with intermittent orchestrated discharges spreading across the whole cluster (**Figure** **3**a; Figure S5a, Supporting Information). These synchronized signals, previously reported as “neural avalanches”,^[^ ^24^, ^25^ ^]^ were further analyzed at the mesoscopic scale (Video S2, Supporting Information). The calcium trace of a representative region in the 3D neuMatrix is shown in Figure 3b,c. Neural clusters in this image were classified into 5 groups according to their spatial distribution and calcium signal. Within each group, the calcium discharges were mostly synchronized with high correlation coefficients (Figure 3d,e), indicating strong local neural connections, as described in Figure 2. Moreover, simultaneous discharges were also observed between groups of neural clusters with distances as great as 2 mm (Figure 3c, indicated by arrows). Another example of 3D neuMatrix with synchronic oscillation across a millimeter scale was illustrated in Figure S6 (Supporting Information). Together with the structural basis of long‐distance neural projections, these results supported the formation of a unique functional large‐scale neural network within the 3D neuMatrix, which we mainly focused on in the subsequent investigations.

We further analyzed the calcium signal of 3D neuMatrix from DIV 1 to DIV 14 (Figure 3f–h) and found that the average firing rate increased with the in vitro culture time but slightly decreased at DIV 14, which was consistent with the cell viability curve (Figure 3f,h). The percentage of clusters that were detected of calcium discharge followed a similar trend (Figure 3f). The average global correlation, defined as the average correlation coefficient between all firing clusters continued to increase over 14 days (Figure 3g,h). Based on the high cell viability, the development of structural and functional neural networks, and the low time cost, we selected DIV 7 as the time point for further studies.

Furthermore, we treated the 3D neuMatrix with antagonists of neurotransmitter receptors to explore the transient effect of small molecules on functional neural networks via calcium oscillation. We used bicuculline (BIC, antagonist of GABA‐A receptor), NBQX (antagonist of AMPA receptor), picrotoxin (PTX, antagonist of GABA receptor), and AP5 (antagonist of NMDA receptor) to selectively disrupt excitatory or inhibitory synapses. Inhibition of GABA receptors with BIC or PTX significantly increased the average firing rate and the percentage of firing clusters in 3D neuMatrix (Figure 3i,j; Figure S5b, Supporting Information). In contrast, inhibition of glutamate receptors with NBQX or AP5 significantly decreased the average firing rate (Figure 3k,l; Figure S5b, Supporting Information). The percentage of firing clusters in a 5‐min duration changed accordingly in PTX and AP5 but not in BIX or NBQX (Figure 3i–l). The average global correlation showed a decreasing tendency in BIC (Figure S5b,c, Supporting Information), NBQX (Figure S5b,c, Supporting Information), and PTX (Figure S5b,d, Supporting Information), but not in AP5 (Figure S5b,d, Supporting Information). This could be attributed to the smaller number of firing clusters after AP5 treatment. These results demonstrated that both excitatory and inhibitory neurons participated in the formation of neural networks in the 3D neuMatrix and that the balance of excitatory neurons (ENs) and inhibitory neurons (INs) contributed to the maintenance of synchronization. Moreover, the 3D neuMatrix exhibited complex functional responses to small molecules, making it suitable for CNS disease modeling and drug screening.

### 2.4 Recreation of Cortical Transcriptomic Profiles in the 3D neuMatrix

**Figure 4:**
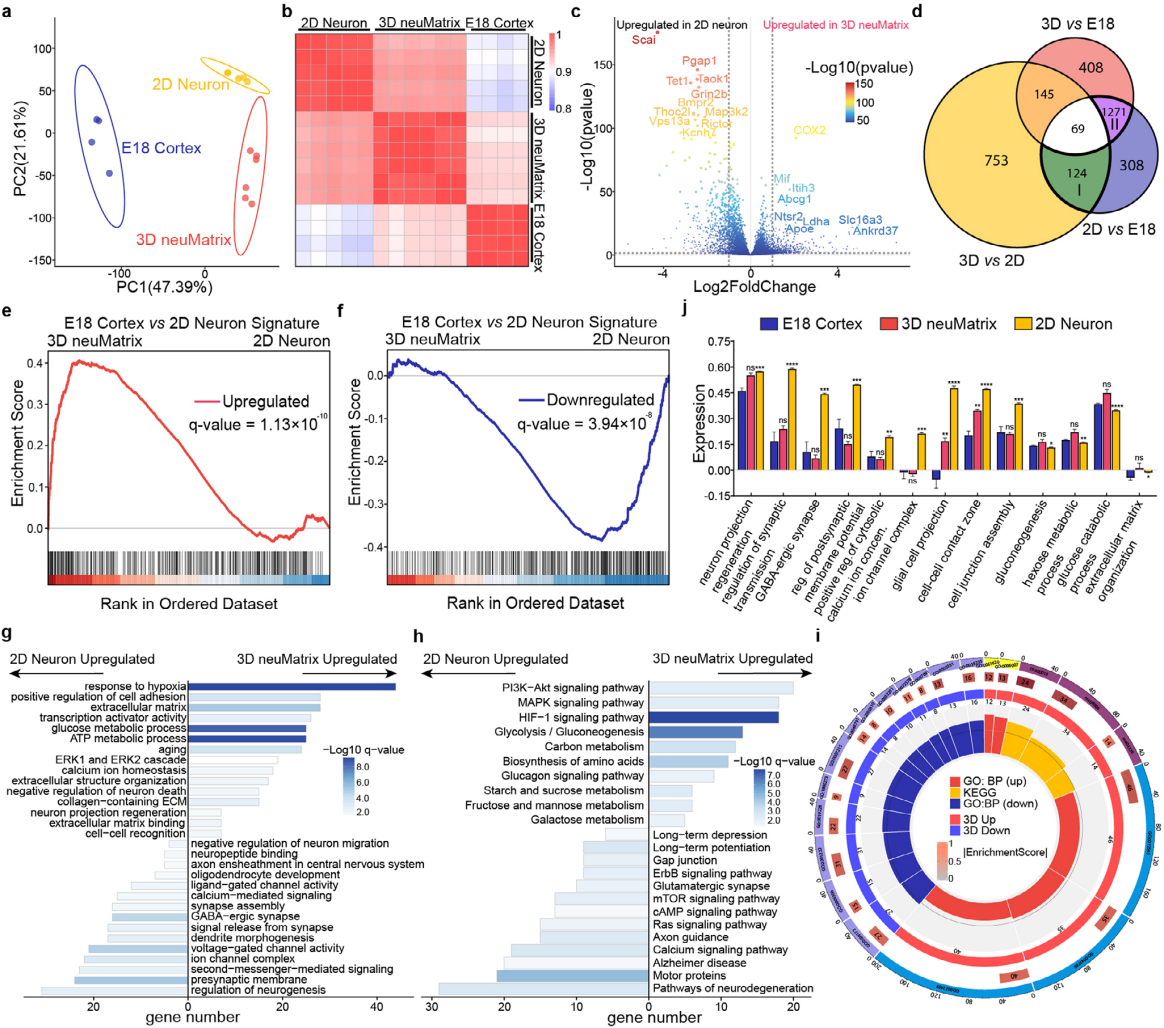
Validation of the gene expression fidelity of 3D neuMatrix by RNA‐seq. a) PCA of RNA‐seq data from the E18 cortex, 2D neuron, and 3D neuMatrix. b) Heatmap displaying global mRNA expression correlations of different models. c) Volcano plot of transcriptional landscapes comparing 3D neuMatrix and 2D neuron. d) Venn diagram showing DEGs in pairwise comparisons of 2D neuron, 3D neuMatrix, and E18 cortex. e) GSEA of the E18 cortex upregulated gene signature when applied to RNA‐seq data comparing 3D neuMatrix with 2D neuron. q‐value = 1.13×10^−10^. f) GSEA of the E18 cortex downregulated gene signature when applied to RNA‐seq data comparing 3D neuMatrix with 2D neuron. *q*‐value = 3.94×10^−8^. g, h) GO (g) and KEGG pathway (h) enrichment analysis of the DEGs between 3D neuMatrix and 2D neuron. i) GSEA of representative differentially enriched gene sets between 3D neuMatrix and 2D neuron. j) Expression of representative pathways related to neuronal function, cellular interactions, and metabolic processes in different models as defined by GSVA. The error bars represent the s.e.m., and the P value was calculated by Welch's *t*‐test. ns, not significant; ^*^ *P* < 0.05; ^**^ *P* < 0.01; ^***^ *P* < 0.001; ^****^ *P* < 0.0001.

It has been extensively characterized that 3D bioprinted cells could more effectively restore the transcriptional profile of parental tissues, compared with 2D or spheroid cultured cells.^[^ ^11^, ^12^, ^26^ ^]^ However, the biological fidelity of 3D bioprinted neural tissue in terms of transcriptional profiles has not been validated in previously published works.^[^ ^15^, ^16^, ^17^ ^]^ Therefore, we performed bulk transcriptional profiling by RNA sequencing (RNA‐seq) on 2D cultured neurons, 3D neuMatrix, and cerebral cortex from E18 rats (Table S1, Supporting Information). Principal component analysis (PCA) revealed different patterns of global transcriptional profiling among three tissues (**Figure** **4**a). The overall correlation of the global expression profile between 3D neuMatrix and E18 cortex was greater than that between 2D neuron and E18 cortex (Figure 4b), indicating the similarity between 3D neuMatrix and E18 cortex. Differentially expressed genes (DEGs) were identified via pairwise comparisons of 2D neurons, 3D neuMatrix, and E18 cortex (Figure 4c,d; Figure S7a,b, Supporting Information). Core sets of upregulated and downregulated genes in E18 cortex versus 2D culture, which were defined as “E18 cortex upregulated” and “E18 cortex downregulated” gene signatures, respectively. Gene set enrichment analysis (GSEA) revealed that compared with those in 2D neurons, the E18 cortex upregulated gene signature was significantly upregulated in 3D neuMatrix, with a normalized enrichment score (NES) of 0.41 (Figure 4e), and the E18 cortex downregulated gene signature was significantly downregulated, with an NES of −0.39 (Figure 4f). These results indicated that 3D neuMatrix could better recapitulate the transcriptional states presented in the cerebral cortex of E18 rats.

To clarify the difference in the transcriptional profile between 2D neuron and 3D neuMatrix, we performed Gene Ontology (GO) and Kyoto Encyclopedia of Genes and Genomes (KEGG) enrichment analyses on the DEGs and conducted GSEA on typical terms (Figure 4g–i). Pathways activated in the 3D neuMatrix were related to cellular interaction, ECM organization, and carbon metabolism. In contrast, 2D neurons displayed an upregulation of pathways related to calcium signaling, neurogenesis, and neurodegeneration. To explore the enrichment of pathways among the three tissue types, we performed gene set variation analysis (GSVA) and found that the overall enrichment scores were more comparable between 3D neuMatrix and E18 cortex (Figure S7c, Supporting Information). Compared with those of 2D neurons, 3D neuMatrix displayed similarities to the E18 cortex in terms of neuron projection, synaptic transmission, and cell–cell interaction signatures (Figure 4j), further supporting the high biological fidelity of the 3D neuMatrix. We speculate that the relatively low enrichment of neural pathways in both 3D neuMatrix and E18 cortex compared with 2D neurons was due to complexity in cellular components, which cannot be fully perceived by bulk analysis.

Moreover, we performed GO and KEGG enrichment analyses on gene subset I (124 genes in the green area), as shown in Figure 4d. 3D neuMatrix was closer to the E18 cortex than 2D neurons with characteristics including neuronal synapses, ECM‐receptor interactions, gated channel activity, and learning‐ or memory‐related pathways (Figure S7d,e, Supporting Information). Similarly, GO and KEGG enrichment analysis of gene subset II (1271 genes in the purple area) in Figure 4d indicated the pathways cerebral cortex had differentially enriched compared to in vitro models, such as neuron projection guidance, regulation of angiogenesis, developmental maturation, and neuron fate commitment (Figure S7f,g, Supporting Information). The 3D neuMatrix had better biological fidelity in terms of global expression profiles and some core sets of E18 cortex‐specific genes and pathways. Taken together, 3D neuMatrix closely resembled in vivo tissue in terms of morphology, function, and molecular signatures.

### 2.5 The 3D neuMatrix Recapitulates the Cellular Components of the Cerebral Cortex

**Figure 5:**
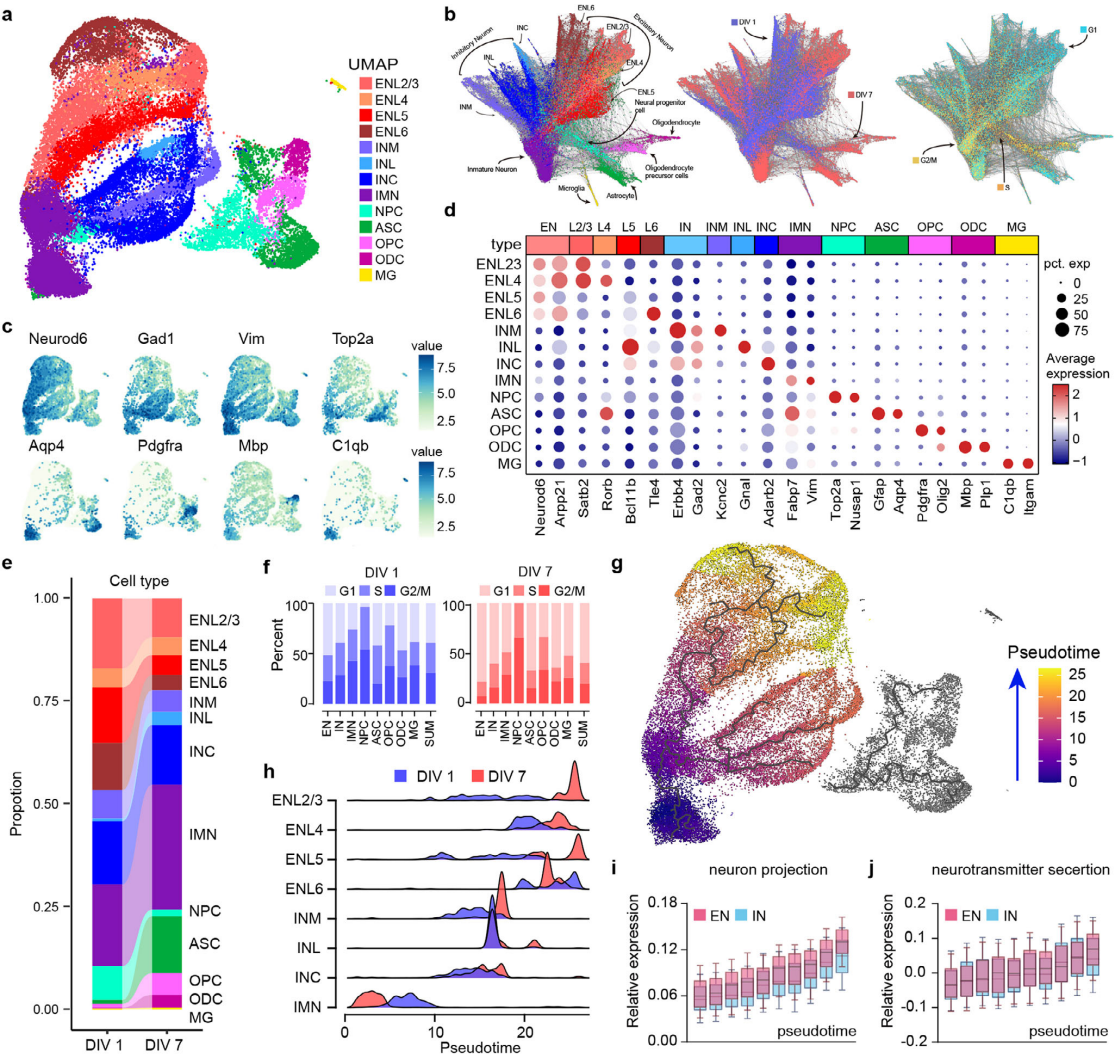
snRNA‐seq of 3D neuMatrix at DIV 1 and DIV 7. a) Projection of all cells plotted by UMAP with cell types indicated by colors. b) Trajectory reconstructed by SPRING with color‐coding cell types (left), timepoints (middle), and cell cycle states (right). c) UMAP plot colored according to the expression of marker genes. d) Dot plot showing the expression of the indicated genes for each cell type. e) Proportion of each cell type at DIV 1 and DIV 7. f) Proportion of cell cycle states (G1, S, G2/M) within each cell type at DIV 1 and DIV 7, with cell cycle states in all cells at the respective time point on the right. g) Pseudo‐time and developmental trajectory calculated by Monocle3 plotted on UMAP. h) Distribution of each type of neural cell along the pseudo‐time axis at DIV 1 and DIV 7. i, j) Box plots showing the sum expression levels of genes specifically annotated by one of four GO terms in the ENs and INs, including neurotransmitter secretion (i) and neural projection (j), along the pseudo‐time axis. ENs, excitatory neurons; INs, inhibitory neurons; IMNs, immature neurons; NPCs, neural progenitor cells; ASCs, astrocytes; OPCs, oligodendrocyte progenitor cells; ODCs, oligodendrocytes; MG, microglia; ENL23, EN layer 2/3; ENL4, EN layer 4; ENL5, EN layer 5; ENL6, EN layer 6; INM, medial ganglionic eminence; INL, lateral ganglionic eminence; INC, caudal ganglionic eminence.

Since bulk RNA‐seq cannot fully restore the complete picture, further analysis at single‐cell resolution is required. To survey the cellular components and time‐related changes within the 3D neuMatrix, we performed single‐nucleus RNA‐seq (snRNA‐seq) at DIV 1 and DIV 7 (**Figure** **5**a,b; Figure S8a,b and Table S2, Supporting Information). A total of 36304 cells (DIV 1, *n* = 20196; DIV 7, *n* = 16108) were extracted. The cell types were annotated by the corresponding markers listed in the Experimental Section (Figure 5a,c,d; Table S3, Supporting Information). To verify the annotation of cells in the 3D neuMatrix, we first compared the annotation results obtained through different clustering methods. The annotations of neural subclusters were consistent with the laminar distribution of ENs and INs through UMAP and SPRING.^[^ ^27^ ^]^ Next, we combined our data with snRNA‐seq data of a neonatal rat cortex from the Gene Expression Omnibus (GEO) (GSE185538) and found that the classification of cells in 3D neuMatrix was robust even when combined with data from the rat cortex (Figure S8c, Supporting Information), verifying the reliability of our annotation. The proportions of different cell types were almost parallel between 3D neuMatrix and in vivo cells (Figure S8d, Supporting Information), indicating that 3D neuMatrix can faithfully recreate the cellular components of the cerebral cortex.

To explore the cellular transformation during culture, we compared the DIV 1 and DIV 7 and found that the cell type compositions were in accordance with the developmental trend in vivo (Figure 5e; Figure S8e, Supporting Information), especially in glial cells with significantly fewer NPCs (8.24% vs 1.61%) and more ASCs (0.93% vs 13.86%) and ODCs (0.15% vs 3.98%) at DIV 7. In neurons, the percentage of INs was mostly stable during in vitro culture (22.91% vs 22.97%), but this did not apply to ENs (46.64% vs 22.34%), which can be mostly attributed to a decrease in the deep layer ENs (ENL5 and ENL6, 25.01% vs 8.51%). The balance between ENs and INs was proposed to be the main factor underlying the functional features, as increased inhibition might induce elevated synchronization,^[^ ^24^, ^25^ ^]^ which is consistent with the increased correlation of calcium oscillation during culture. To further analyze the proliferative capacity, cell cycle analysis was conducted and revealed a reduced percentage of cells in the S state (29.77% vs 20.64%) and G2/M state (30.80% v 19.79%) overall as well as in each cell type separately (Figure 5b,f), demonstrating a diminished dividing capacity at DIV7. To further illustrate the transcriptomic changes during in vitro culture, GO and KEGG terms were enriched by DEGs between DIV 1 and DIV 7 in each cell type, and GSVA results of representative terms were illustrated in Figure S8f (Supporting Information). All cell types showed upregulation in functions of neural networks, including the development of dendrites and axons, synaptic organization and signaling, and downregulation in overall RNA and protein synthesis. Interestingly, glial cells exhibited increased gliogenesis and myelination, consistent with previous results on the formation of glia‐facilitated neural cluster structures (Figure 2) and an increase in differentiated glial cells (Figure 5e) at DIV 7.

As 3D neuMatrix depicts the maturation of cell types and elevated expression of differentiated functions, we conducted pseudo‐time analysis to further illustrate the developmental process in the 3D neuMatrix. The pseudo‐time trajectory calculated by Monocle3 was aligned with the UMAP projection showing the developmental direction from DIV 1 to DIV 7, from immature neurons (IMNs) to differentiated neurons (Figure 5g). All clusters of neurons exhibited a further distribution along the pseudo‐time trajectory at DIV 7, except for IMNs (Figure 5h), despite having fewer dividing cells at DIV 7 (Figure 5b,f). These IMNs appeared to shift from the dividing state to a more primal dormant state during in vitro culture. To our knowledge, the IMNs in our study exhibited high expression of Fabp7 and Vim, which are both markers of radial glia during neurodevelopment,^[^ ^28^, ^29^ ^]^ though radial glia were not identified via snRNA‐seq of the rat cortex. To determine the nature of these IMNs, we examined the expression of several feature genes (Figure S8g, Supporting Information). These IMNs exhibited relatively low expression of stemness markers reported in the radial glia of humans.^[^ ^30^ ^]^ Meanwhile, these cells expressed high levels of pan‐neuron markers but not mature neuron markers.^[^ ^29^, ^31^ ^]^ Together, the characteristics of these neurons match those of immature neurons, and more research on the snRNA‐seq of rat cortical cells should be conducted. For mature neurons, we selected 4 neuron‐related GO terms to examine the development of neural functions along the pseudo‐time axis (Figure S8h, Supporting Information). An increasing trend of enrichment in neuron projection and neurotransmitter secretion along the pseudo‐time axis was observed (Figure 5i,j). The enrichment of synapse assembly remained relatively high, and the enrichment of neural differentiation remained at the same level since primary cortical neurons were mostly differentiated (Figure S8i,j, Supporting Information). Overall, these results support previous findings on the structural and functional complexity of the 3D neuMatrix, emphasizing the ability of 3D neuMatrix to restore multiple features of the cerebral cortex.

### 2.6 Modeling of Ischemic Stroke using the 3D neuMatrix

**Figure 6:**
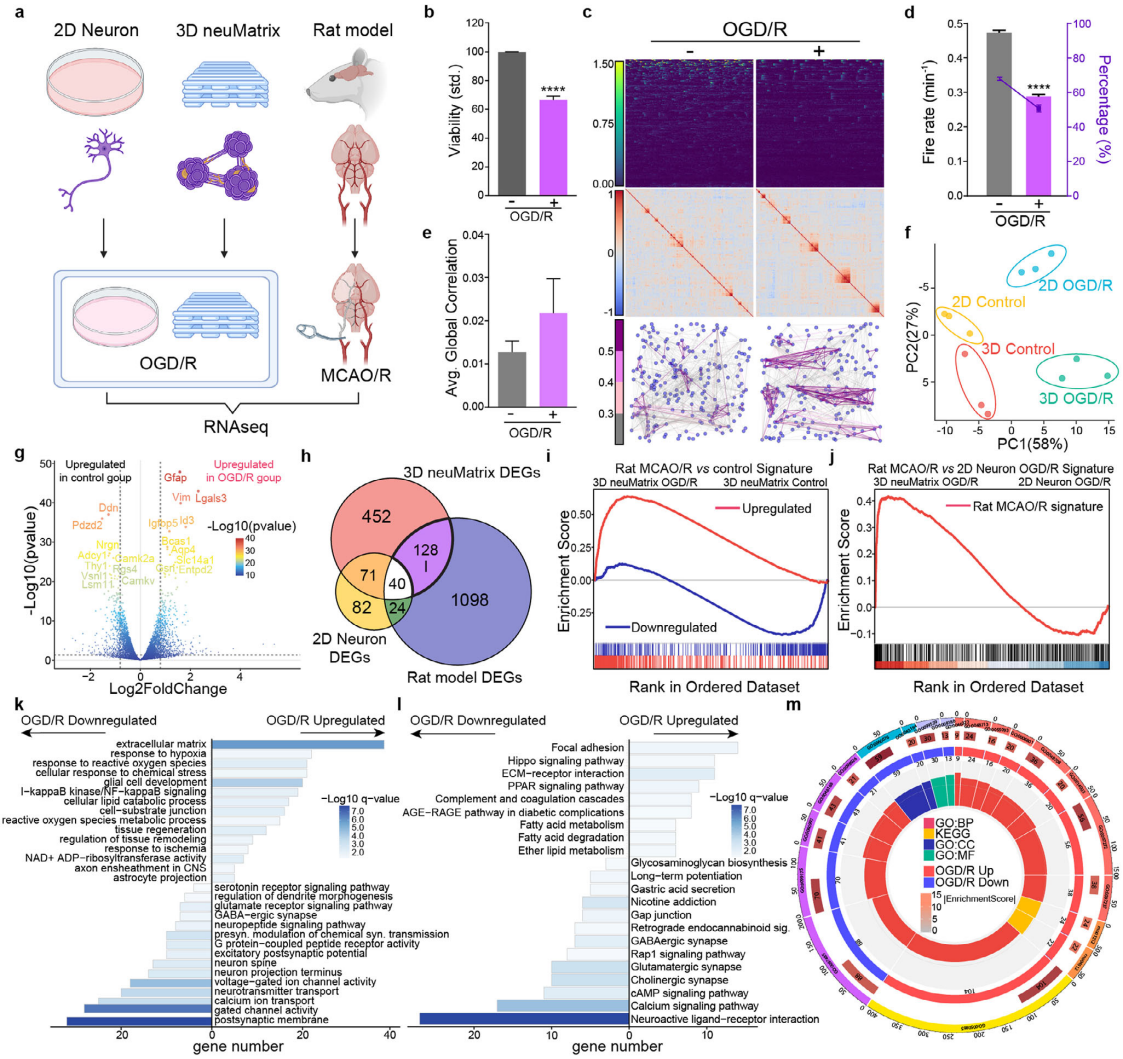
OGD/R modeling of the 3D neuMatrix with restoration of features of ischemic stroke. a) Illustrating a diagram of the methods of stroke modeling in different modalities. b) Viability of the 3D neuMatrix in the control and OGD/R groups were measured by ATP quantification (*n* = 3). c) Representative images of the firing matrix (upper), correlation matrix (middle), and correlation diagram (lower) in the control and OGD/R groups. d, e) Average firing rate and percentage of firing neural clusters (d) and average global correlation (e) in the control and OGD/R groups (*n* = 4). f) PCA of RNA‐seq data from models of ischemic stroke using E18 cortex, 2D neuron, and 3D neuMatrix. g) Volcano plot of the transcriptional landscapes of 3D neuMatrix in the control and OGD/R groups. h) Venn diagram showing DEGs in pairwise comparisons of different models of ischemic stroke. i) GSEA of the “rat MCAO/R upregulated” and “rat MCAO/R downregulated” gene signatures when applied to the RNA‐seq data comparing 3D neuMatrix in the control and OGD/R groups. *q*‐value < 10^−10^. j) GSEA of the rat MCAO/R‐specific gene set when applied to the RNA‐seq data comparing the OGD/R models of 2D neuron and 3D neuMatrix. *q*‐value < 10^−10^. k, l) GO (k) and KEGG pathway (l) enrichment analysis of the DEGs between the control and OGD/R groups in 3D neuMatrix. m) GSEA of representative differentially enriched gene sets between the control and OGD/R groups in 3D neuMatrix. The error bars represent the s.e.m., and the P value was calculated by Welch's t‐test. ^****^ *P* < 0.0001.

To evaluate the ability of 3D neuMatrix in disease modeling and drug screening, we selected ischemic stroke as the representative disease and treated the 3D neuMatrix and 2D neuron with oxygen‐glucose deprivation/reperfusion (OGD/R) to recreate the stroke process (**Figure** **6**a). We replaced the medium of 3D neuMatrix with low‐glucose DMEM and placed it hypoxia chamber for respective times of 1, 2, 3, or 4 h, followed by 24 h of incubation in complete Neurobasal medium and normoxia. Compared with that of the control group, cell viability was significantly reduced after 2 h of OGD, and after 4 h of OGD, the relative viability was reduced to 49.0 ± 0.6% (Figure 6b; Figure S9a, Supporting Information). We have chosen a 4‐h OGD for subsequent analysis. For calcium oscillation, the average firing rate and percentage of firing cells both significantly decreased after OGD/R treatment (Figure 6c,d), though the average global correlation showed an increased tendency (Figure 6c,e).

To verify the modeling efficacy of 2D neuron and 3D neuMatrix, we performed RNA‐seq on the two models under normoxic and OGD/R conditions and compared them with the transcriptome of a rat MCAO/R model from the GEO dataset (GSE163614). PCA revealed similar alteration in the transcriptome of 2D neuron and 3D neuMatrix between the normoxia and OGD/R groups (Figure 6f; Table S4, Supporting Information). DEGs between the normal and disease groups were identified for each modality (Figure 6g,h; Figure S9d, Supporting Information). GSEA revealed that compared to those in the control group, the OGD/R model of 3D neuMatrix displayed significant upregulation of the rat MCAO/R upregulated gene set, and downregulation of the rat MCAO/R downregulated gene set (Figure 6i). When compared to the OGD/R model of 2D neurons, the 3D neuMatrix showed significant upregulation of the rat MCAO/R‐specific gene set (Figure 6j). These results indicated that the OGD/R model of 3D neuMatrix can better replicate the transcriptional characteristics of ischemic stroke in vivo.

Subsequently, to evaluate pathway regulation in the OGD/R models of 3D neuMatrix and 2D neurons, we examined the enrichment of GO and KEGG terms, and GSEA was conducted (Figure 6k–m). In the OGD/R model of 3D neuMatrix, response to hypoxia and ischemia, ECM interactions, glial cell development, and lipid metabolism processes were significantly enriched, suggesting that the 3D neuMatrix could respond to OGD/R in key molecular features. In the OGD/R model, 3D neuMatrix displayed downregulation of pathways related to ligand‒receptor interactions, gated channels, and synapse components, which is consistent with the neuronal damage caused by an ischemic stroke. In the OGD/R model of 2D neurons, GO analysis and GSEA indicated that the upregulated pathways were mainly related to cell cycle regulation, wound healing, and ECM organization (Figure S9e, Supporting Information). Although both 2D neuron and 3D neuMatrix exhibited downregulation of neural function‐related pathways in the OGD/R model, the upregulated pathways in 3D neuMatrix more accurately simulated the response of neural tissue to oxidative stress and reperfusion injury in ischemic stroke. Finally, to explore which pathways in the OGD/R model of 3D neuMatrix more closely resemble those in the in vivo model compared to 2D neuron, we performed GO and KEGG enrichment analyses on gene subset I, as shown in Figure 6h (128 genes). We found that the OGD/R model of 3D neuMatrix could better recapitulate the characteristics of important ischemic stroke‐related pathways, such as response to stimuli and hypoxia, tissue remodeling, and response to axon injury (Figure S9g,h, Supporting Information).

Additionally, the 3D neuMatrix could serve as a drug screening platform. For the treatment of ischemic stroke, two drugs, edaravone, and butylphthalide, which have been approved for the treatment of ischemic stroke in Japan and China, respectively, were selected.^[^ ^32^, ^33^ ^]^ Unfortunately, the addition of these two drugs did not improve cellular viability after OGD/R (Figure S9b, Supporting Information) or functional performance, as indicated by the firing rate and percentage of firing cells (Figure S9c, Supporting Information). We speculated that 4 h of OGD causes irreversible damage to neurons, as is the case in clinical settings.^[^ ^34^ ^]^ More candidate compounds could be tested to explore potential treatments for ischemic stroke.

In summary, the 3D neuMatrix, an emerging 3D culture technology, overcomes the limitations of in vitro and in vivo models. This technique utilizes bioprinting technology to achieve precise control of experimental settings while maximally preserving the in vivo biological environment. As a next‐generation research model, the 3D neuMatrix shows tremendous potential in neuroscience, disease research, and drug screening.

## 3 Discussion

We used 3D bioprinting to fabricate an in vitro neural model, the 3D neuMatrix. This in vitro model only takes 7 days to establish a complex and reproducible neural network with multi‐scale functional connections. Calcium signal recording enabled the visualization of spontaneous discharges within neural clusters, as well as synchronized calcium signals at the millimeter scale, which is similar to the functional architecture of cortical columns and projections in the brain. Moreover, the gene expression profiles of 3D neuMatrix were more similar to those of the E18 cortex than were those of the 2D‐cultured neurons under both normal and disease conditions, further highlighting the fidelity of 3D neuMatrix. snRNA‐seq revealed that the 3D neuMatrix recreated the cellular components of the cerebral cortex, with neurodevelopmental changes during in vitro culture. Overall, the 3D neuMatrix provides a valuable in vitro research platform for studies on neural circuit mechanisms, CNS disease mechanisms, drug screening, and tissue engineering.

In vitro modeling of CNS is key to understanding the pathophysiological process and development of therapies. Conventional 2D monolayer culture failed to recapitulate the intricate cell‐cell and cell‐matrix interactions or the microenvironment in vivo. 3D scaffolds composed of rigid and flexible biomaterials have been employed to support neuronal growth and network formation.^[^ ^35^, ^36^, ^37^ ^]^ However, neuronal interconnections in the scaffold predominantly localize to the surfaces, with limited biological fidelity.^[^ ^7^ ^]^ Other alternative approaches include neurospheres by cell aggregation,^[^ ^38^, ^39^ ^]^ yet this method lacks precise spatial control over cellular organization, leading to inter‐batch heterogeneity.^[^ ^40^ ^]^ Cerebral organoids and region‐specific brain organoids aid in elucidating brain development, disease mechanisms, and neural circuit dynamics.^[^ ^8^, ^41^, ^42^ ^]^ However, brain organoids are not terminally differentiated and often require extended culture time up to several months.^[^ ^43^, ^44^ ^]^ Brain‐on‐chip microfluidic systems offer refined regulation over microenvironments and synaptic networks,^[^ ^45^, ^46^ ^]^ but face challenges in the design of a physiologically relevant model and the requirement of specialized manufacturing.^[^ ^47^ ^]^ Nevertheless, our 3D neuMatrix platform achieves high‐throughput construction with desirable reproducibility while enabling precise spatial organization of neural networks, addressing critical gaps in existing neural modeling platforms.

The 3D neuMatrix, a next‐generation in vitro model for neural cells, offers several advantages. First, we optimized the bioprinting process for neural tissue engineering using primary cortical cells. As terminally differentiated neurons are delicate and susceptible to manipulation,^[^ ^48^ ^]^ several studies have employed induced pluripotent stem cells (iPSCs)‐derived neural progenitors to bioprint complex neural constructs since these cells are more resilient to physical stimulation during printing and can be induced to form region‐specific neurons.^[^ ^49^ ^]^ For example, neural progenitors of the upper and deep layers can be used to restore the laminar cortical structure,^[^ ^50^ ^]^ or neurons can be mixed with astrocytes,^[^ ^13^ ^]^ immune cells,^[^ ^51^ ^]^ or endothelial cells^[^ ^52^ ^]^ to recreate different aspects of functional neuron units. However, iPSC‐derived neurons face the hindrance of maturation and therefore cannot fully represent the characteristics of neurons in vivo. Through a combination of our techniques and the versatility of bioprinting, many complex models of the CNS with high fidelity could be created, including the model of Parkinson's disease using primary neurons from the cortex, striatum, and thalamus^[^ ^53^ ^]^ or by adding glioma cells to study glioma‐neuron interactions.^[^ ^54^ ^]^ Second, the 3D neuMatrix exhibited multiscale functional neural connections, which were observed at the micrometer and millimeter scales. Interestingly, the inter‐cluster network is unique to bioprinting models, as direct inoculation of bioinks without the printing process exhibited neural cluters without large‐scale interconnections (Figure S2g, Supporting Information). This feature could be contributed to the predefined spatial cues provided by the 3D bioprinting, which can be further incorporated with the scalability of bioprinting to construct even larger neural tissues by design (Figure 1b), potentially repairing the physically damaged neural tissue with personalized structure and restoring neural functions.^[^ ^50^, ^55^ ^]^ Recent developments in in vitro intelligence have integrated cultured neuron models (usually cerebral organoids) and brain‐computer interfaces to harness the computational power of in vitro neural networks.^[^ ^19^, ^20^ ^]^ As the 3D neuMatrix possesses much more complex functional structures, our model could potentially advance biocomputing complexity with the integration of 3D sensory in the future.^[^ ^56^ ^]^ Third, the 3D neuMatrix is suitable for disease modeling and has the potential to simulate various pathological conditions. Small molecules can be screened by 3D neuMatrix high‐throughput for treatment efficacy, and their viability, function, and transcriptome can be evaluated using standard laboratory equipment.^[^ ^57^ ^]^ Combined with gene editing technology, bioprinted tissue has the potential to replicate complex neural circuits among different brain regions and related neurodegenerative diseases, such as Parkinson's disease, Alzheimer's disease, rare genetic metabolic diseases such as Alexander disease, and amyotrophic lateral sclerosis.^[^ ^58^ ^]^

Some limitations exist in our prototype bioprinted neural tissue. The bioink we have selected is gelatin‐based, which cannot fully restore the ECM of the brain. Hyaluronic acid (HA) and laminin, being one of the major components in nervous tissue, promote neural maturation and neurite projections, and have been widely used in bioprinting of neural stem cells and neural tissue repair.^[^ ^59^, ^60^, ^61^ ^]^ Currently, there is few literatures using HA or laminin to culture primary neurons, most were scaffold‐based with inferior neural network structure.^[^ ^62^, ^63^, ^64^ ^]^ Further optimization of incorporating HA and laminin in bioink is critical to establish a more physiologically relevant and functional neural construct, including the molecular weight and modification of HA, gelation process, and suitable rheological properties for bioprinting of matured neurons.^[^ ^65^, ^66^, ^67^ ^]^ As we fabricated the 3D neuMatrix by extrusion‐based printing in a layer‐by‐layer fashion, shear force damage could occur during the printing process. In addition, the increase in the diameters of neural cell clusters during in vitro culture could lead to insufficient oxygen and nutrient infiltration and induce hypoxic reactions in the centers of cell clusters. These limitations could be countered by the use of emerging biofabrication technologies such as acoustic tweezers or the incorporation of vascularized structures. Acoustic tweezers utilize ultrasonic waves to manipulate the position and movement of biocomponents.^[^ ^68^ ^]^ One of the key advantages of acoustic tweezers is their contactless fabrication procedure, which allows the manipulation of cells without causing mechanical damage.^[^ ^69^ ^]^ Moreover, as we removed meninges in the preparation of cortical cells, 3D neuMatrix contains few endothelial cells and no vasculature, which limits our simulation of ischemic stroke to direct damage on neurons only, but not the whole pathophysiology. Constructing vascularized structures shows promise for improving nutrient and oxygen delivery and the scalability of tissue fabrication.^[^ ^70^ ^]^ ETV2, a transcription regulator, has been transfected into endothelial cells in several studies to increase vasculature formation in bioprinting systems.^[^ ^21^, ^71^ ^]^ Networks of interconnected channels within 3D bioprinted tissue could also be created by a void‐free printing approach based on a sacrificial templating phase.^[^ ^72^ ^]^ Future adaptations, such as incorporating vascularization or the blood‐brain barrier, are needed to enhance our model for more authentic simulations.

## 4 Conclusion

In conclusion, we assembled an in vitro neural tissue named 3D neuMatrix, with defined cell components and neural circuits as well as regulatable spatial organization made possible by 3D bioprinting. Single‐cell analysis revealed the differentiation of neuron subtypes and glial cells, with elevated expression in neural communication during in vitro culture. Disease modeling with the 3D neuMatrix confirmed its potential in creating more physiologically relevant models for pathogenesis and therapeutic research on CNS disorders.

## 5 Experimental Section

### Isolation and 2D Culture of Primary Cortical Neurons

Primary neurons were derived from the cerebral cortex of Sprague–Dawley (SD) rats at E18. Briefly, pregnant SD rats were terminally anesthetized by isoflurane in a chamber. Embryos were taken out and brains were dissected and placed in ice‐cold high‐glucose Dulbecco's Modified Eagle's Medium (DMEM; Gibco), and the meninges were removed carefully. Cortices were cut into 0.5 mm^3^ pieces and digested with 2 mg mL^−1^ papain and 0.2 mg mL^−1^ DNase for 30 min at 37 °C. Primary cortical cells were released by gentle trituration and sieved through a 70 µm mesh. For 2D cultures, cortical cells were resuspended in DMEM supplemented with 10% fetal bovine serum (Gibco), plated at a density of 5×10^4^ cm^−2^ in 3.5 cm dishes precoated with poly‐D‐lysine (Gibco), and incubated at 37 °C and 5% CO_2_. The medium was changed to complete Neurobasal medium (Gibco) supplemented with 1×B27 (Gibco), 1 mm GlutaMax (Gibco), 50 U mL^−1^ penicillin, and 50 µg mL^−1^ streptomycin (Gibco) 3 h after plating.

### 3D Bioprinting of Primary Cortical Neurons

GelMA with a 30% degree of methacrylation (GelMA30; EFL) was dissolved in phosphate‐buffered saline (PBS) to 12% (w/v) and sterilized with a 0.22 µm filter (Millex, Merck) for stock solution. Isolated cortical neural cells were resuspended in a complete Neurobasal medium. For formulation of the bioink for printing, the cell suspension was mixed with preheated 12% (w/v) GelMA30 and 1% (w/v) lithium phenyl‐2,4,6‐trimethylbenzoylphosphinate at a ratio of 4:5:1 to a final concentration of 1.5×10^7^ cells mL^−1^ in 6% (w/v) GelMA30. The mixed bioink was transferred to a sterilized syringe equipped with a 21G needle, which was then loaded into the 3D bioprinter at a predesigned temperature. The temperatures of the nozzle and the forming chamber were fine‐tuned to identify the ideal conditions that preserved the highest cell viability during the printing process. The bioprinting process utilized an extrusion‐based bioprinter (SUNP BIOTECH) following a previously established protocol. Briefly, the gross shape of 3D neuMatrix was designed as a square cube with independent channels between the frames, which could maximize the contact between the primary neural cells and the medium. The 3D bioprinter was used to continuously fabricate 3D neuMatrix by extrusion in a layer‐by‐layer fashion within each well of a 24‐well plate (Corning). The printing speed was set to 4.8 mm s^−1^, and the extrusion speed was set to 1.5 mm^3^ s^−1^. Subsequently, the 3D neuMatrix was exposed to 405 nm light of 7.5 mW cm^−2^ for 10 s to facilitate crosslinking and then supplied with 1 mL of complete medium and maintained at 37 °C with 5% CO_2_. The whole bioprinting procedure took ≈1 h. Neuronal maturation was induced by 7 days of in vitro culture, and half of the medium was refreshed every 2 days, followed by subsequent assessments.

For the fabrication of an in vitro mini‐brain, the floating bioprinting technique, which was developed by Zhuoran Jiang in his unpublished work, was utilized. Briefly, 12.5% (w/v) Pluronic F127 (Sigma–Aldrich) was dissolved in PBS at 4 °C overnight before 2.5% (w/v) H‐HPMC (Sangelose 90L, Daido Chemical Co.) was added to the Pluronic F127 solution with vigorous stirring. The mixture was centrifuged at 1000 rpm for 1 min to remove bubbles and carefully transferred into boxes as printing chambers. Then, 5% (w/v) α‐cyclodextrin (Sigma–Aldrich) was dissolved in PBS with vigorous stirring as the washout solution. 3D models of the brain were generated in 3D Builder (Microsoft) as built‐in models. The samples in the STL format were saved and loaded onto a bioprinter, which was subsequently bioprinted as described previously. The bioprinted mini‐brain construct was transferred to a 6‐well plate with a‐CD solution for washout of extra Pluronic F127.

### Cell Viability

The proliferation of 3D neuMatrix was analyzed by Live/Dead assay and ATP quantification (CellTiter‐Glo; Promega) at D1, D4, D7, and D10. Briefly, the 3D neuMatrix was washed with PBS and stained with 1 µmol L^−1^ calcein‐AM (Sigma) and 2 µmol L^−1^ propidium iodide (Sigma) for 15 min at room temperature in the dark. Then, the 3D neuMatrix was washed with PBS before being observed under a laser scanning confocal microscope (C2/C2si; Nikon). For ATP quantification, the culture medium was removed, and CellTiter was added with fresh medium at a ratio of 1:1. The culture plate was incubated for 25 min at 80 rpm, and the supernatant was transferred to a 96‐well plate and read by a multimode microplate reader.

### SEM

The 3D neuMatrix was fixed with 2.5% glutaraldehyde before being snap‐frozen in slush nitrogen for 30 s, and the acellular GelMA construct was directly snap‐frozen. The samples were maintained at low temperature and under vacuum and transferred to a preparation chamber for sublimation at −90 °C and sputter coating with gold. The samples were imaged with a scanning electron microscope (FEI Quanta 450) at −140 °C.

### Rheology and Mechanical Behavior of Bioinks

The storage modulus (G′) and loss modulus (G″) of the bioink under different shear strains were measured by rheometer (MCR 302; Anton Paar). The viscosity of bioink under different shear stress was measured by a precision universal tester (AGS‐X‐50N; Shimadzu).

### Immunofluorescence (IF)/Immunohistochemical Staining

The 3D neuMatrix was washed with PBS before being fixed with 4% paraformaldehyde for 30 min at room temperature. For permeabilization and blocking, 3% bovine serum albumin in 0.1% Triton X‐100 was used, and 3D neuMatrix was incubated for 1 h at room temperature and 60 rpm. Primary antibodies were diluted with 0.1% Triton X‐100, and the 3D neuMatrix was further incubated at 4 °C overnight. The 3D neuMatrix was washed with PBS 3 times before being incubated with secondary antibodies diluted with 1% bovine serum albumin in the dark for 2 h. After PBS washes, DAPI and phalloidin‐FITC (P5282; Sigma–Aldrich) were added, and the cells were incubated for 30 min. The 3D neuMatrix was rinsed in PBS and observed under a laser scanning confocal microscope (A1R; Nikon). Imaris software (Oxford instrument) was used for the reconstruction of 3D neuMatrix. Ki67 was quantified by ImageJ (Fiji) with a threshold of 40 in intensity and a minimum size of 15 µm^2^. Antibodies employed in this study are listed: Mouse anti‐NeuN (ab104224; Abcam), Rabbit anti‐MAP2 (ab32454; Abcam), Mouse anti‐Nestin (ab6320; Abcam), Rabbit anti‐Tuj1 (ab18207; Abcam), Rabbit anti‐GFAP (GB11096‐100; Servicebio), Rabbit anti‐Ki67 (ab15580; Abcam); Goat anti‐mouse IgG AlexaFluor488 (ab150113; Abcam); Goat anti‐rabbit IgG AlexaFluor594 (ab150080; Abcam)

### Calcium Signal Analysis

The 3D neuMatrix was washed with HBSS and incubated with Fluo‐4 AM (Thermo Fisher) for 30 min at 37 °C. The 3D neuMatrix was transferred to a 35 mm confocal dish with a preheated complete neurobasal medium and observed under a laser scanning confocal microscope (A1R; Nikon) in a stage‐top incubator (Tokai Hit). Five‐minute time‐lapse sequences were taken for each study, with a maximum of 3 levels on the z‐axis to reflect 3D neural signals. For drug treatment, image sequences were taken as controls before neurotransmitter receptor antagonists were added to the medium in a confocal dish to their destined concentrations. After 5 min of reaction and stabilization, image sequences were taken for the treatment group.

Image sequences were preprocessed by FluoroSNNAP^[^ ^73^ ^]^ and Suite2P^[^ ^74^ ^]^ for large‐scale and small‐scale imaging, respectively. Putative neural clusters were selected by threshold selection on the average image, and the fluorescence intensity was extracted for each neural cluster. The ΔF/F_0_ calculation and further analysis were completed with customized Python scripts. F_0_ was defined as the mean fluorescence intensity of 10 consecutive frames with the lowest intensity. For each frame, if its ΔF/F_0_ was greater than the mean ΔF/F_0_ of all frames plus 3 standard deviations, it was defined as the frame with neural firing. The firing rate for each neural cluster was calculated as the percentage of frames with neural firing in all frames.

For analysis of the correlation between each neural cluster, Pearson's correlation coefficients of ΔF/F_0_­were computed. Clusters with no firing across all frames were excluded to avoid false high correlations.

### Next‐Generation Sequencing Data Generation and Analysis

The 3D neuMatrix and E18 cortex were transferred to cryogenic tubes, immediately frozen with liquid nitrogen, and then stored at −80 °C. For RNA isolation and qualification, the frozen tissue samples were placed in a precooled mortar and ground to powder. The tissue was then treated with TRIzol (Invitrogen, CA, USA) and RNase‐free DNase I (TaKaRa, Kusatsu, Japan). For 2D cultured neurons, cells were directly treated with TRIzol and RNase‐free DNase I to extract RNA. The RNA purity and concentration were evaluated using 1% agarose gels and a NanoDrop spectrophotometer (Thermo Scientific, DE, USA). An Agilent 2100 Bioanalyzer (Agilent Technologies, CA, USA) was used to assess RNA integrity. Sequencing libraries were generated using the NEBNext Ultra RNA Library Prep Kit for Illumina (NEB, USA) following the manufacturer's recommendations, and index codes were added to attribute sequences to each sample. The purified libraries were analyzed with an Illumina HiSeq 6000 sequencer with 150‐bp paired‐end reads. Fastp was used to obtain clean reads by removing adapter‐containing reads, poly‐N‐containing reads, and low‐quality reads. Subsequent analyses were conducted solely on high‐quality clean reads. Read mapping was conducted using the rat reference genome through STAR (v.2.7.2a). HTSeq v 0.5.4 p3 was utilized to count the reads mapped to each gene. Gene expression levels were estimated using the fragments per kilobase of transcript per million fragments mapped (FPKM) method. Subread feature Counts and FPKM were used for gene‐level quantification.

Differential expression analysis between two conditions/groups was conducted using the DESeq2 R package^[^ ^75^ ^]^ (version 1.40.2). In the differential analysis between the 2D neuron and the 3D neuMatrix, “Average log2FoldChange larger than 1 or less than −1” and “adjusted P value less than 0.05” were considered to indicate statistical significance. In the comparison between 2D neuron and the E18 cortex, as well as in the differential analysis between the 3D neuMatrix and the E18 cortex, genes with “Average log2FoldChange larger than 1.5 or less than −1.5” and “adjusted P value less than 1 × 10e^−10^” were considered differentially expressed. GO/KEGG enrichment analysis was performed using the clusterProfiler package (v.4.0.5). Subsequently, representative GO/KEGG terms and gene signatures were chosen for GSVA and GSEA utilizing the GSVA package^[^ ^76^ ^]^ (v.1.48.3) and clusterProfiler^[^ ^77^ ^]^ package (v.4.0.5), respectively.

### snRNA‐seq Generation and Preprocessing

At DIV 1 and DIV 7, the 3D neuMatrix was transferred to cryogenic tubes and rapidly frozen in liquid nitrogen. Nuclei were extracted with a chromium nuclei isolation kit (PN‐1000494, 10x Genomics), and nuclei suspensions were loaded onto 10× Genomics Chromium platforms according to the manufacturer's protocol. snRNA‐seq libraries were generated and sequenced on an Illumina NovaSeq 6000 platform. The raw reads were demultiplexed and aligned to NCBI_mRatBN7.2 via the 10× Genomics Cell Ranger V7.0.1 pipeline using default parameters. The Seurat package (v.4.3.0) was used for downstream analysis. Quality control was performed by deleting nuclei expressing fewer than 200 genes or more than 10% unique molecular identifiers (UMIs) associated with mitochondrial genes. “NormalizeData” and “ScaleData” were used to generate the expression matrix of the remaining nuclei. Nuclei expressing more than one major cellular marker were considered doublets and removed from each cluster. The remaining single nuclei that passed the filtering criteria were used for subsequent analysis.

### Unsupervised Clustering and Identification of Cell Subpopulations

After identifying the main cell population through first‐run clustering, the Seurat pipeline was ran for a second time. In this study, the unwanted effects caused by the percentage of mitochondrial UMI were eliminated by regression. A resolution of 0.5 and the top 50 PCs of the PCA were used to divide the cell population. Harmony was used with the default settings for additional batch correction. The cell types were annotated by the expression of their corresponding markers. Due to the limited research on single‐cell annotation of the rat cortex, cell type‐specific markers were selected based on three studies of the rat cortex^[^ ^78^, ^79^, ^80^ ^]^ and well‐recognized nervous system markers in humans.^[^ ^29^, ^30^, ^31^ ^]^ The expression of the following genes was used to annotate the cell types: Neurod6 and Arpp21 for ENs; Erbb4, Gad2, and Gad1 for INs; Fabp7 and Vim for IMNs; Nusap1 and Top2a for NPCs; Aqp4, Slc1a2, and Gfap for ASCs; Cspg4, Pdgfra, and Olig2 for OPCs; Plp1, Ugt8, and Mbp for ODCs; and C1qb, Ccl4, and Itgam for MGs. ENs were further classified into corresponding cortical layers, with expression of Cux1 and Satb2 for EN layer 2/3 (ENL23), Rorb for EN layer 4 (ENL4), Bcl11b for EN layer 5 (ENL5), and Tle4 and Foxp2 for EN layer 6 (ENL6). INs were subclustered by precursors with expression of Sox6 and Kcnc2 for the medial ganglionic eminence (INM), Gnal and Dach1 for the lateral ganglionic eminence (INL), and Adarb2 and Dlx1 for the caudal ganglionic eminence (INC). To further reduce the dimensionality of the dataset, UMAP or t‐distributed stochastic neighbor embedding (tSNE) was used to visualize the clustering results. SPRING was also used^[^ ^27^ ^]^ to ensure that the cell classification was robust. Public snRNA‐seq data (GSE185538) of the neonatal rat cortex were obtained from the NCBI GEO for comparative analysis of cell type annotations. The data were processed in the same way as described above. Figure S4e (Supporting Information) shows the distribution and stability of 3D neuMatrix cell type annotations across different datasets.

### Monocle3 Pseudo Time Analysis

Monocle3 v1.0.0 was used to characterize the developmental trajectory of the cells. The pseudo‐time trajectory was inputted by the learn_graph call with use_partition = F. Cells were then ordered in pseudo‐time, and differential gene expression testing was performed to identify cluster‐specific markers and gene modules using the find_gene_modules function. Visualizations were produced using built‐in Monocle3 functions.

### Differential Expression Analysis and GSVA

The FindAllMarkers() function of Seurat was utilized to identify marker genes that were significantly overexpressed in different clusters. For analysis of the significance of each gene between two different clusters, “average log2FoldChange larger than 0.5 or less than −0.5” and “adjusted P value less than 0.05” were considered to indicate statistical significance for subsequent analysis. GO/KEGG enrichment analysis was conducted using the clusterProfiler package (v.4.0.5). Representative terms were selected for GSVA among all cell subtypes using the GSVA package (v.1.48.3). Significance was assessed by the Wilcoxon rank‐sum test and the BH procedure.

### OGD/R

The culture medium of the 3D neuMatrix and 2D cultured neuron was replaced with low‐glucose DMEM (Gibco) immediately before the neurons were placed in a hypoxia chamber pumped with 95% N2 and 5% CO_2_. After the respective hours of OGD, the culture medium was changed back to a complete neurobasal medium, and the cells were placed in an incubator with 5% CO_2_ for another 24 h. Cell viability analysis, calcium signal analysis, and RNA extraction were conducted as previously described. For analysis of the efficacy of drugs, low‐glucose DMEM and complete Neurobasal medium supplemented with certain drugs were added during the OGD/R process. The cell viability and calcium signal analysis of each group were compared. For differential expression analysis under OGD/R conditions, an adjusted *P* value < 0.05 and a |log2(foldchange)| > 0.8 were set as the thresholds for significant differential expression in the 2D neuron and 3D neuMatrix model. The GSE163614 dataset was also incorporated as an animal model in comparison to the OGD/R model using 2D neurons and 3D neuMatrix. The brain cortex tissue of SD rats was isolated 24 h after cerebral artery occlusion and reperfusion injury. Next, RNA‐seq was performed to determine differences in gene expression profiles between the treated groups and the sham group. For differential expression analysis in the rat MCAO/R model, an adjusted *P* value < 0.05 and a |log2(foldchange)| > 1 were set as the thresholds for significantly differential expression during data processing.

### Statistics and Reproducibility

No statistical methods were used to determine the sample size, and the experiments were not randomized. The data collection and analysis were not blinded. No data were excluded from the analysis. R (v.4.2.2) was used for the statistical analysis. Significance was assessed by unpaired Student's t‐tests, two‐sided Wilcoxon tests, and Kruskal–Wallis tests. *P* < 0.05 was considered to indicate statistical significance.

### Ethics Declarations

The Institutional Animal Care and Use Committee (IACUC) of Peking Union Medical College Hospital approved all the animal protocols used in this study (XHDW‐2024‐16).

### Data Availability

The code for calcium image analysis can be downloaded from GitHub (https://github.com/ChimesZ/neuMatrix). Other data that support the findings of this study are either included within this article or available from the email of corresponding author, upon reasonable request.

## Conflict of Interest

The authors declare no conflict of interest.

## Author Contributions

H.Y., J.Z., Y.L., Z.Z. and W.L. contributed equally to this work. H.Y. and J.Z. fabricated the 3D neuMatrix with assistance from Huayu Y., N.Y. and W.L. H.Y. and J.Z. performed floating bioprinting with assistance from Z.J. H.Y. and J.Z. performed immunostaining. Z.Z., H.Y. and J.Z. performed calcium signal analysis. Y.L. and J.Z. performed bulk RNA‐seq and snRNA‐seq analysis with assistance from H.Y. and L.O. W.L., J.Z. and H.Y. performed OGD/R modeling with assistance from H.L., Y.L., and N.Y. H.Y., J.Z., Y.L., Z.Z. and W.L. designed the study and wrote the manuscript with support from Huayu Y., Y.W., N.Y., Y.M. and W.M.

## Supporting information



Supporting Information

Supplemental Table 1

Supplemental Table 2

Supplemental Table 3

Supplemental Table 4

Supplemental Video 1

Supplemental Video 2

## Data Availability

The data that support the findings of this study are available from the corresponding author upon reasonable request.
