# Supplementary material for: Multiscale Organization of Neural Networks in a 3D Bioprinted Matrix
Source: Adv Sci (Weinh). 2025 May 28;12(30):e04455. doi: 10.1002/advs.202504455 (PMC12376583; doi:10.1002/advs.202504455)
Supplement: Supplementary file 1 — Supporting Information [file ADVS-12-e04455-s006.docx]

Supporting Information

Multiscale Organization of Neural Networks in a 3D Bioprinted Matrix

**
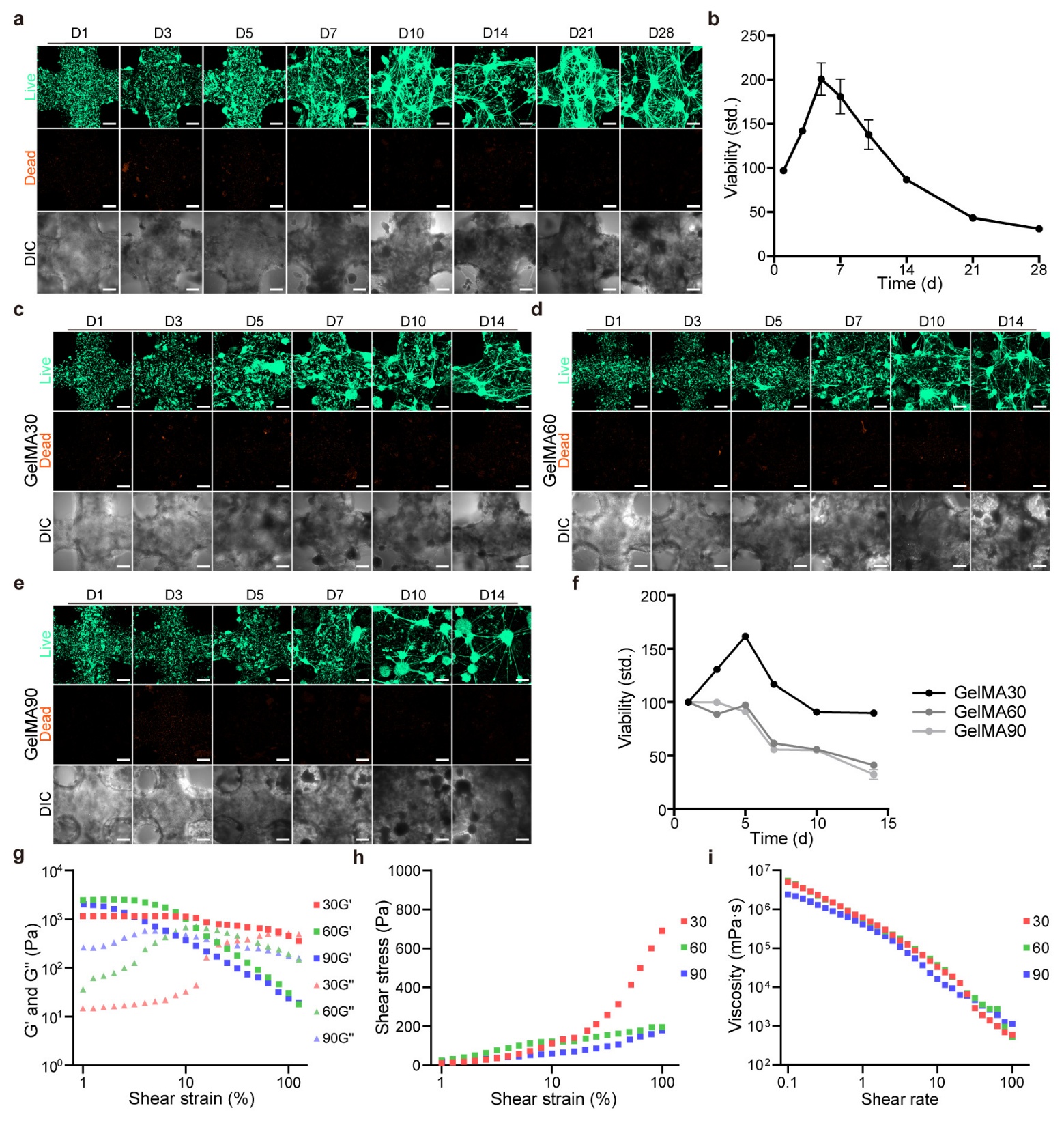
***Huiyu Yang^1,4^, Jiangang Zhang^2,4^, Yiran Li^3^, Zihan Zhong^1,4^, Wenhua Li^5^, Haojun Luo^5^, Yanyong Liu^5^, Liujian Ouyang^6^, Zhuoran Jiang^2^, Yuning Sun^2^, Hang Sun^2^, Lulu Liu^7^, Huayu Yang^2*^, Yu Wang^1*^, Nan Yang^5*^, Yilei Mao^2*^, Wenbin Ma^1*^*

**Figure S1. Optimization of bioink composition. a** Live/Dead staining and differential interference contrast (DIC) image sequences of 3D neuMatrix at DIV 1, 3, 5, 7, 10, 14, 21, 28. **b** Viability of the 3D neuMatrix as determined by ATP quantification during prolonged *in vitro* culture. **c-e** Live/Dead staining and DIC image sequences of 3D neuMatrix constructed by GelMA30 (**c**), GelMA60 (**d**), and GelMA90 (**e**) at DIV 1, 3, 5, 7, 10, 14. **f** Viability of the 3D neuMatrix constructed by GelMA of different degree of substitution (DS) as determined by ATP quantification during *in vitro* culture. **g** Storage (G′) and loss (G″) moduli of GelMA of different DS (30, 60, 90%). **h** Compression representative curves of GelMA of different DS (30, 60, 90%). **i** Viscosity and shear-thinning behavior of GelMA of different DS (30, 60, 90%). Scale bar, 200 μm. The error bars represent the s.e.m..


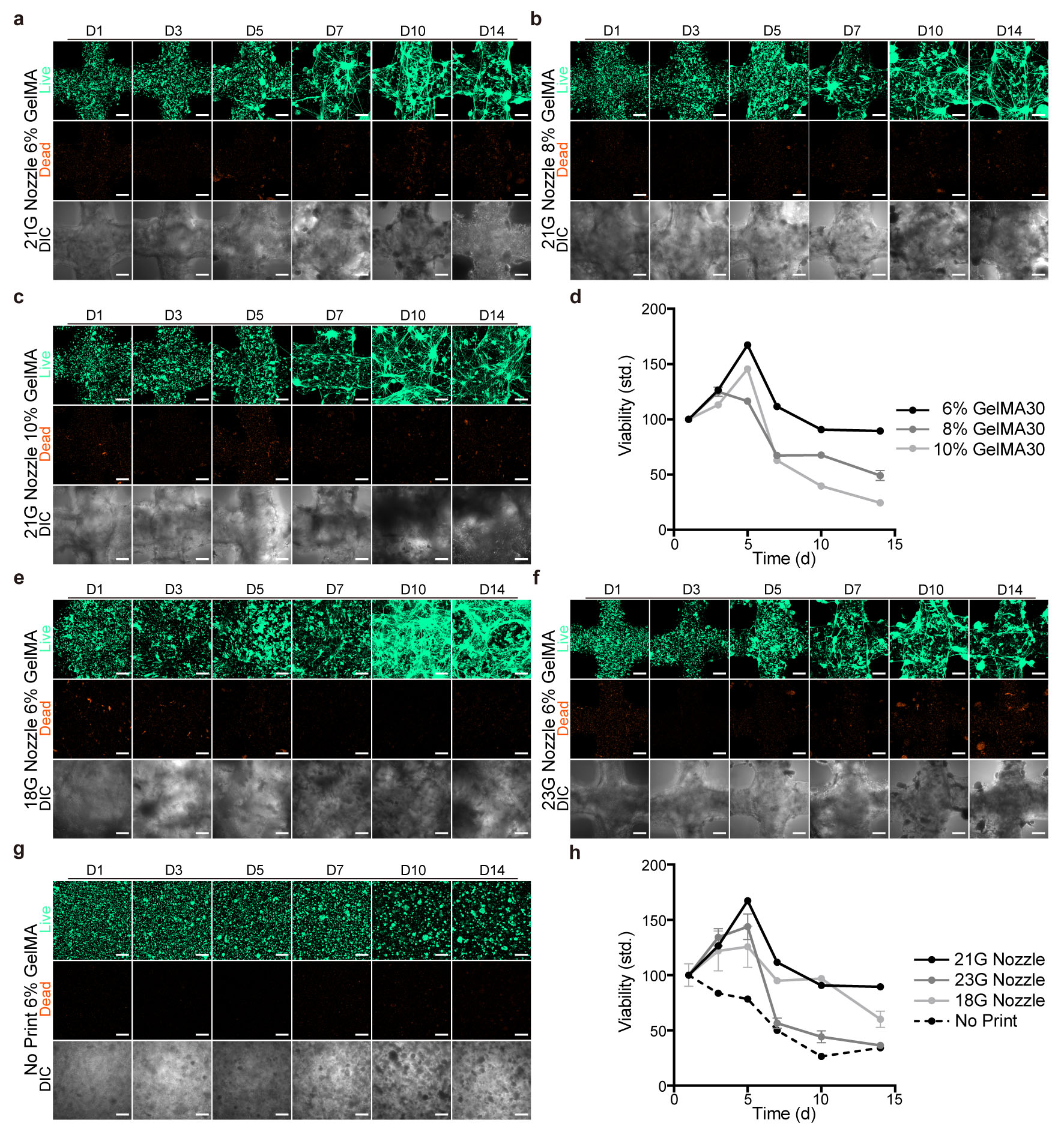
**Figure S2. Optimization of bioink composition and bioprinting conditions. a-c** Live/Dead staining and DIC image sequences of 3D neuMatrix constructed by 21G nozzle and 6% GelMA (**a**), 8% GelMA (**b**), and 10% GelMA (**c**) at DIV 1, 3, 5, 7, 10, 14. **d** Viability of the 3D neuMatrix constructed by different concentrations of GelMA as determined by ATP quantification during *in vitro* culture. **e-g** Live/Dead staining and DIC image sequences of 3D neuMatrix constructed by 18G nozzle (**e**), 23G nozzle (**f**), and no print (**g**) at DIV 1, 3, 5, 7, 10, 14. **h** Viability of the 3D neuMatrix constructed by different nozzles or no printing as determined by ATP quantification during *in vitro* culture. Scale bar, 200 μm. The error bars represent the s.e.m..


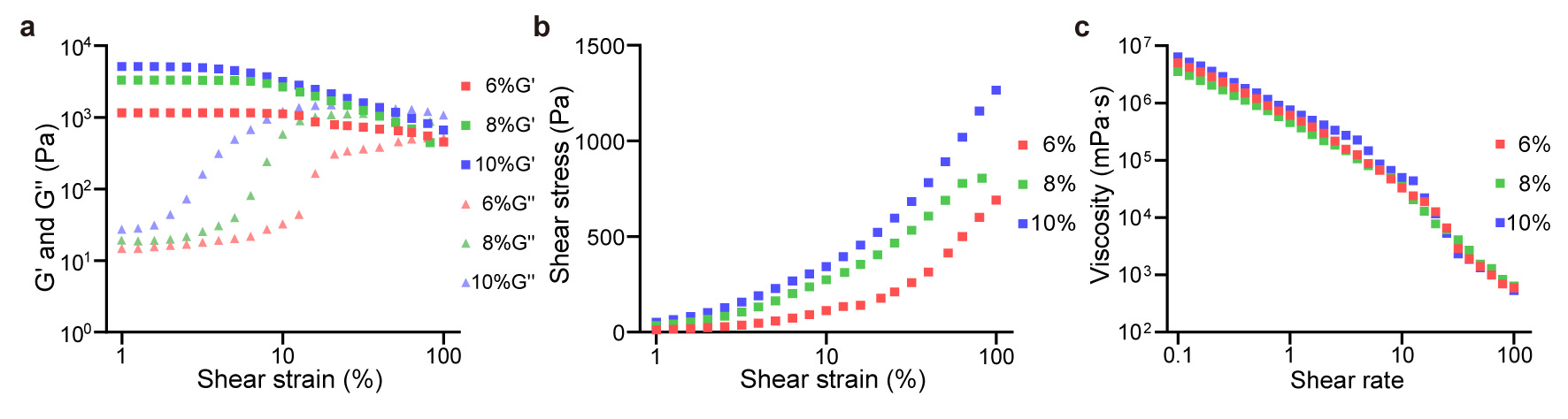
**Figure S3. Rheological properties of GelMA in different concentration. a** Storage (G′) and loss (G″) moduli of GelMA of different concentration (6, 8, 10%). **b** Compression representative curves of GelMA of different concentration (6, 8, 10%). **c** Viscosity and shear-thinning behavior of GelMA of different concentration (6, 8, 10%).

**
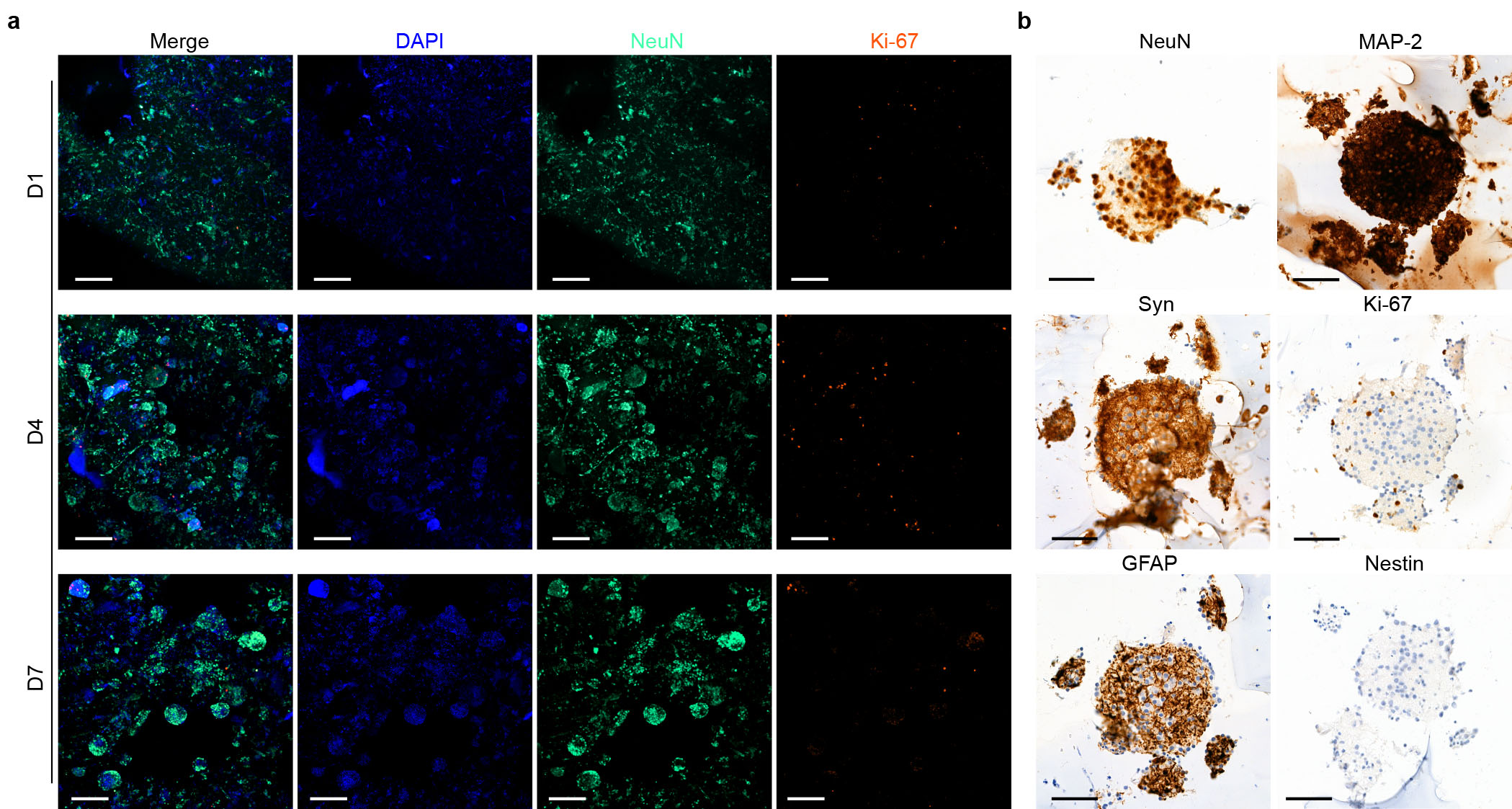
Figure S4. Immunostaining of the 3D neuMatrix. a** IF images of DAPI (blue) for cell nuclei, NeuN (green) for neurons, and Ki67 (red) for proliferating cells at DIV 1, 4, and 7. **b** Immunohistochemistry images of representative neural clusters in the 3D neuMatrix. Scale bar, 200 μm (**a**), 50 μm (**b**).

**
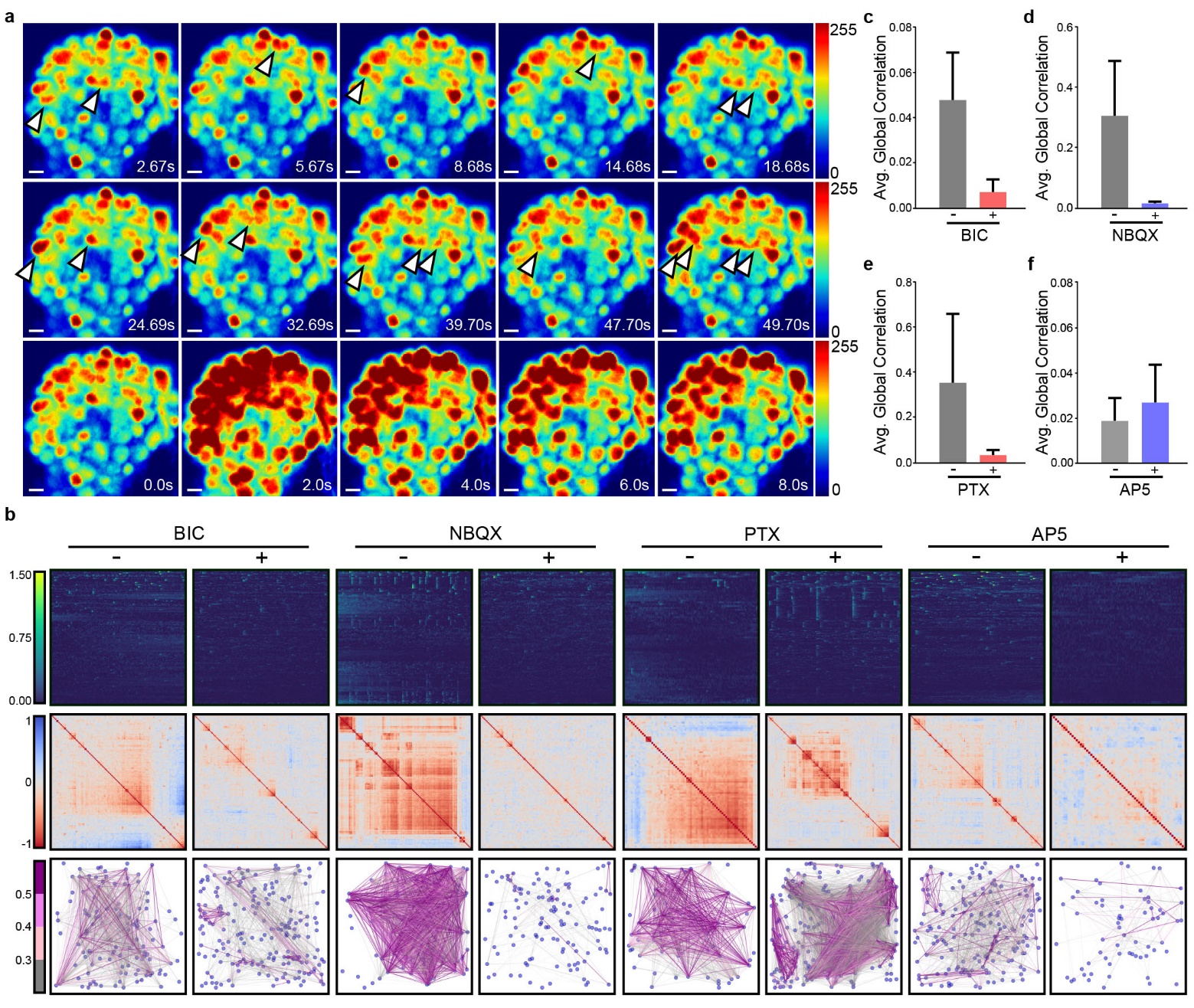
Figure S5. Calcium signaling of the 3D neuMatrix. a** Image sequence of the calcium signal inside a representative neural cluster, with white arrows indicating the onset of neuronal firing (upper and middle) and a synchronized discharge involving the whole cluster. **b** Representative images of the firing matrix (upper), correlation matrix (middle) and correlation diagram (lower) before and after treatment with BIC, NBQX, PTX and AP5. **c-f** Alteration of the average global correlation (n=4) before and after treatment with BIC (**c**), NBQX (**d**), PTX (**e**), and AP5 (**f**). Scale bar, 10 μm. The error bars represent the s.e.m..


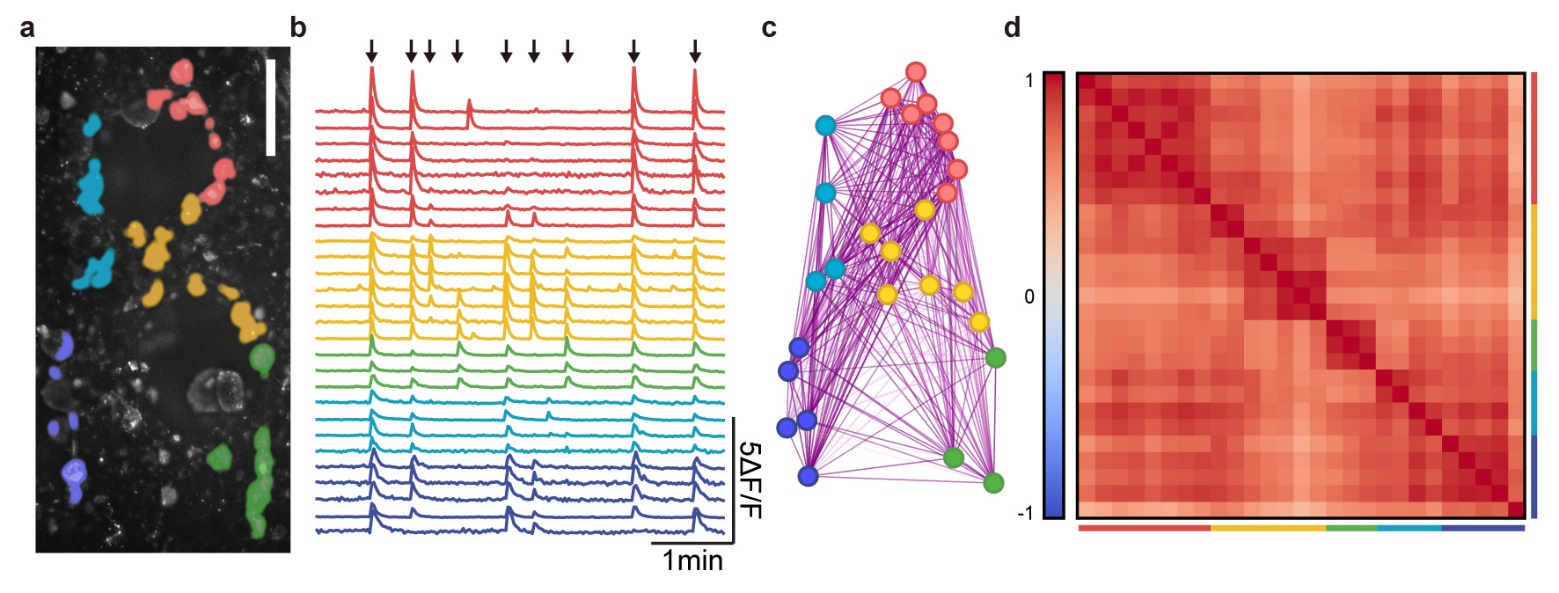
**Figure S6. Long-range functional connections in 3D neuMatrix. a-d** Mesoscopic calcium signal among neural clusters marked in **a**, traces of which are illustrated in **b**, with black arrows indicating synchronized firing involving ≥3 groups of neural clusters. The correlations of signaling between clusters were portrayed *in situ* (**c**) and in matrix (**d**) in the order of upper to lower position within each group of neural clusters in **a**. Scale bar, 500 μm.

**
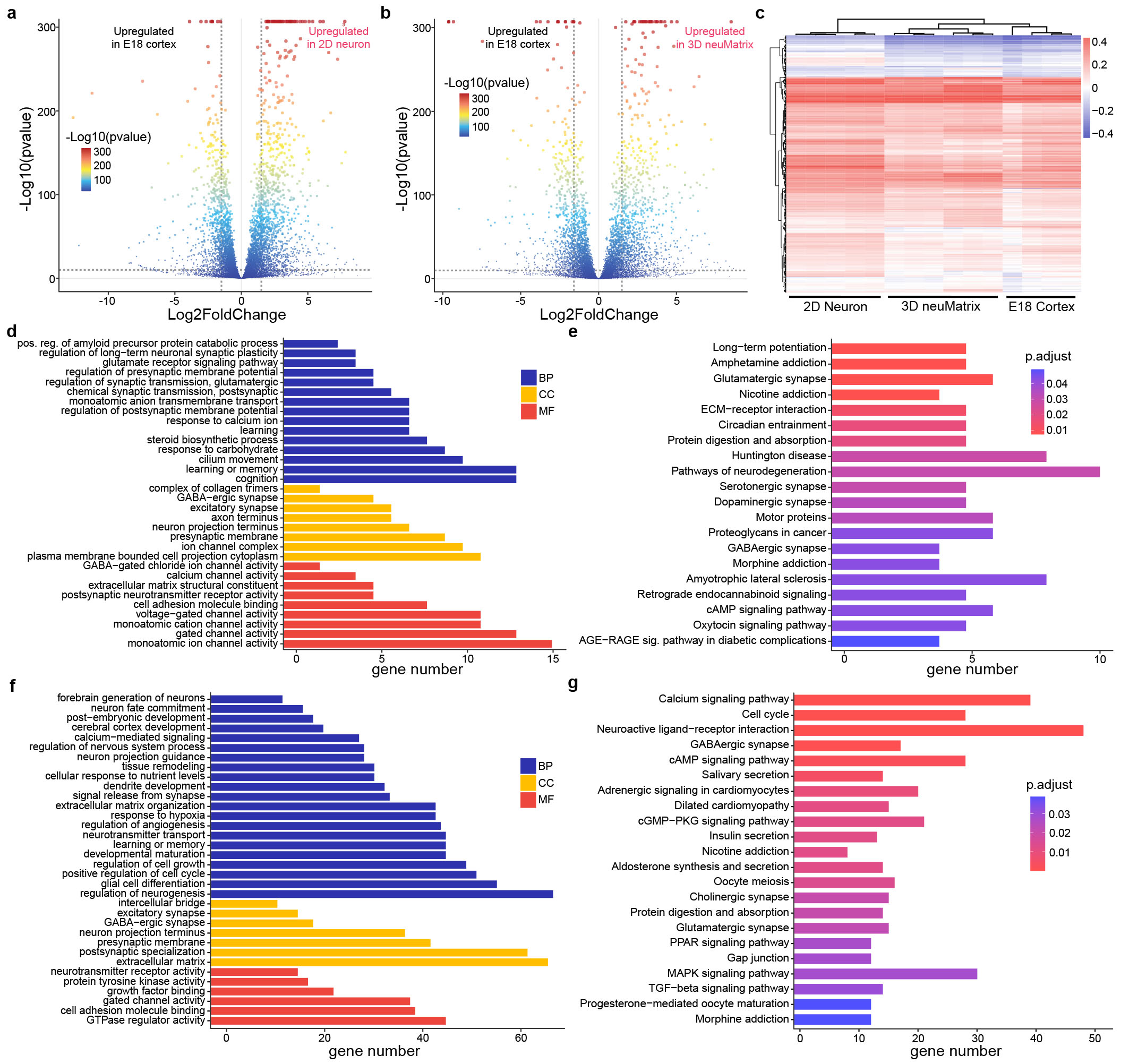
Figure S7. Characterization of transcriptional profiles in the E18 cortex, 2D neuron and 3D neuMatrix. a-b** Volcano plot of transcriptional landscapes comparing the 2D neuron and E18 cortex (**a**), as well as the 3D neuMatrix and E18 cortex (**b**). The x-axis depicts the log-transformed fold change, while the y-axis displays the log-transformed P value adjusted for multiple test correction. **c** Heatmap displaying the expression of differentially enriched GO/KEGG terms between the 3D neuMatrix and 2D neuron in different models as defined by GSVA. **d-e** GO (**d**) and KEGG pathway (**e**) enrichment analysis of gene subset I in Figure 5d (124 genes). **f-g** GO (**f**) and KEGG pathway (**g**) enrichment analysis of gene subset II in Figure 5d (1271 genes).

**
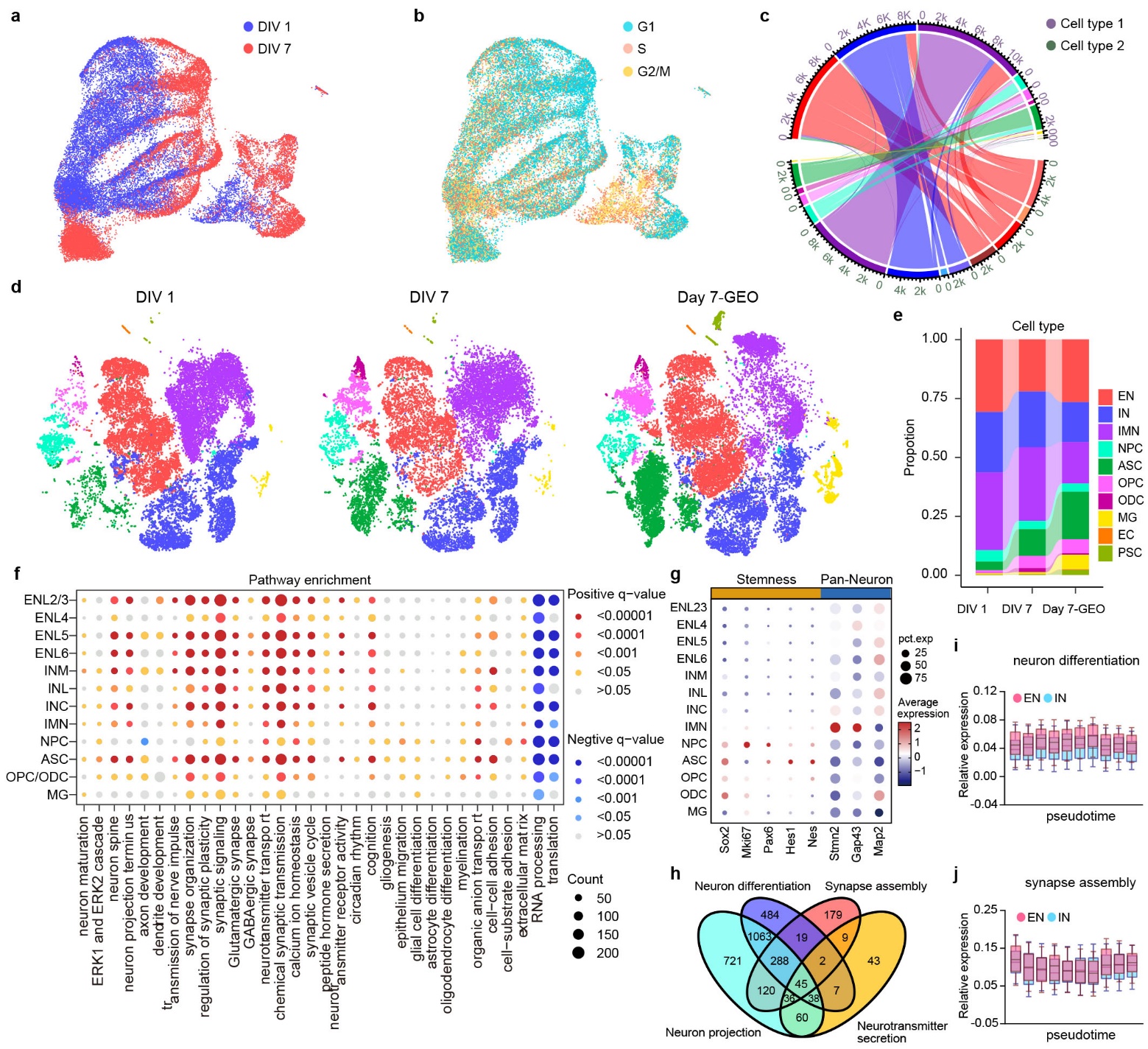
Figure S8. snRNA-seq of the 3D neuMatrix. a-b** snRNA-seq of the 3D neuMatrix at DIV 1 and DIV 7 plotted by UMAP with time points (**a**) and cell cycle states (**b**) indicated by colors. **c** Cell type annotations of the 3D neuMatrix when annotated in combination with snRNA-seq data of the D7 neonatal rat cortex (cell type 1) or alone (cell type 2). **d** UMAP plot of the 3D neuMatrix at DIV 1 (left, n=20196), the 3D neuMatrix at DIV 7 (middle, n=16108) and the D7 neonatal rat cortex (right, n=24518). **e** Proportions of each cell type in the 3D neuMatrix at DIV 1, the 3D neuMatrix at DIV 7 and the D7 neonatal rat cortex. **f** Representative pathways enriched by GSVA at DIV 7 and DIV 1. **g** Dot plot showing the expression of stemness-related and pan-neuron marker genes for each cell type. **h** Venn diagram of genes in four neuron-related GO terms; genes exclusive to each term (721 for neuron projection, 484 for neuron differentiation, 179 for synapse assembly, and 43 for neurotransmitter secretion) were used for subsequent pseudotime analysis. **i-j** Box plots showing the sum expression levels of genes specifically annotated by one of four GO terms in the ENs and INs, including neuron differentiation (**i**) and synapse assembly (**j**), along the pseudotime axis.

**
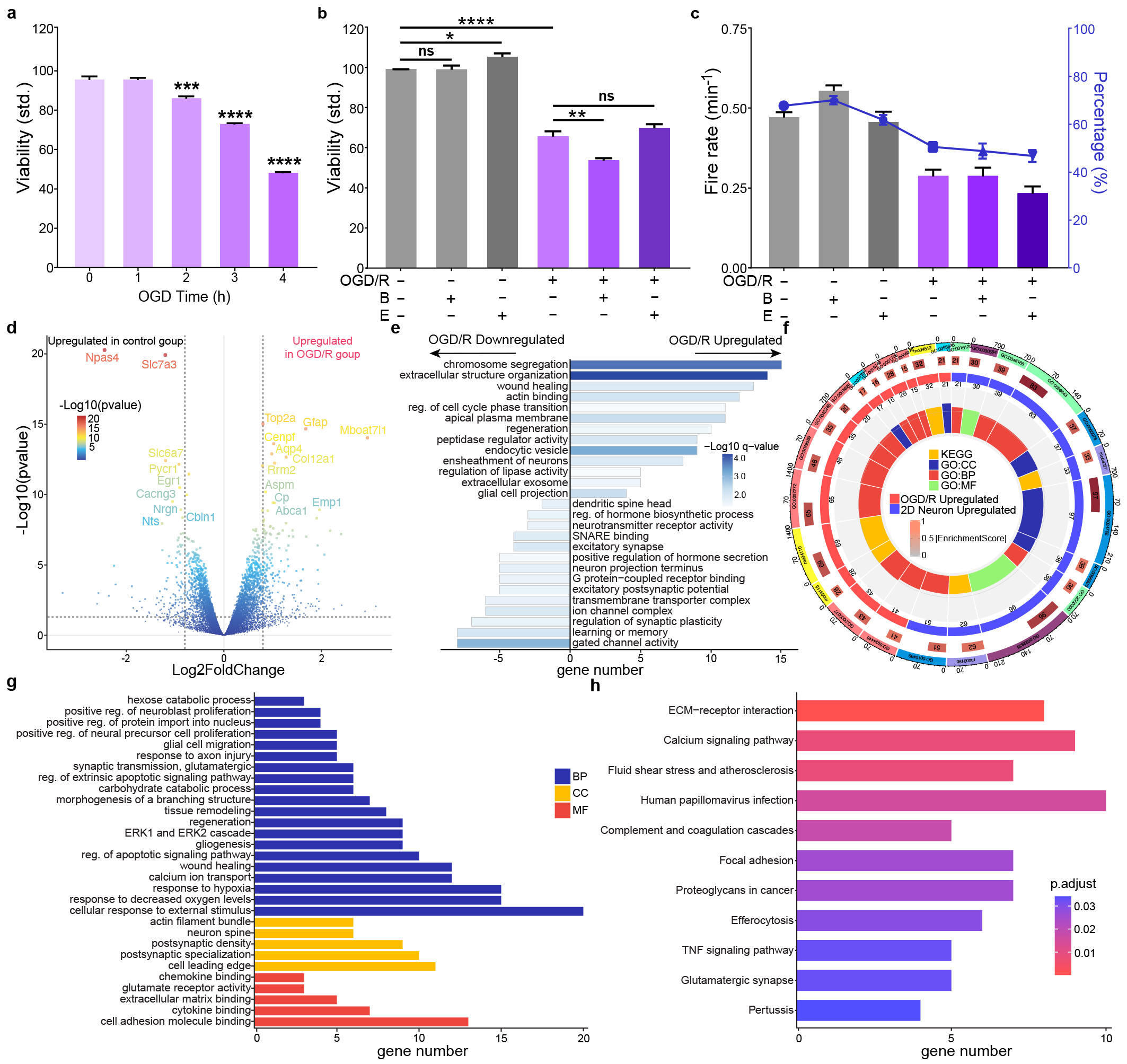
Figure S9. OGD/R modeling of the 3D neuMatrix. a** Viability of the 3D neuMatrix after 0, 1, 2, 3, and 4 hours of OGD treatment followed by 24 hours of reperfusion; 4 hours of OGD were selected for subsequent analysis. **b** Viability of the 3D neuMatrix in the control and OGD/R groups treated with or without butylphthalide (B, 10 μM) or edaravone (E, 10 μM), as determined by ATP quantification (n=3). **c** Average firing rate and percentage of firing neural clusters (n=4) in the control and OGD/R groups with or without B or E. **d** Volcano plot of transcriptional landscapes comparing the control and OGD/R groups in 2D neuron. **e** GO pathway enrichment analysis of the DEGs between the control and OGD/R groups in 2D neuron. **f** GSEA of representative differentially enriched gene sets between the control and OGD/R groups in 2D neuron. **g-h** GO (**g**) and KEGG pathway (**h**) enrichment analysis of gene subset I in Figure 6h (128 genes). The error bars represent the s.e.m., and the P value was calculated by Welch’s t test. ns not significant, **P<0.05, **P<0.01, ***P<0.001, ****P<0.0001.*
